# A Novel Glycoside Hydrolase DogH Utilizing Soluble Starch to Maltose Improve Osmotic Tolerance in *Deinococcus radiodurans*

**DOI:** 10.3390/ijms24043437

**Published:** 2023-02-08

**Authors:** Yuan Gui, Min Lin, Yongliang Yan, Shijie Jiang, Zhengfu Zhou, Jin Wang

**Affiliations:** 1 College of Life Science and Engineering, Southwest University of Science and Technology, Mianyang 621000, China; 2 Key Laboratory of Agricultural Microbiome (MARA), Biotechnology Research Institute, Chinese Academy of Agricultural Sciences, Beijing 100081, China

**Keywords:** *Deinococcus radiodurans*, osmotic stress, trehalose, novel glycoside hydrolase, DogH

## Abstract

*Deinococcus radiodurans* is a microorganism that can adjust, survive or thrive in hostile conditions and has been described as “the strongest microorganism in the world”. The underlying mechanism behind the exceptional resistance of this robust bacterium still remains unclear. Osmotic stress, caused by abiotic stresses such as desiccation, salt stress, high temperatures and freezing, is one of the main stresses suffered by microorganisms, and it is also the basic response pathway by which organisms cope with environmental stress. In this study, a unique trehalose synthesis-related gene, *dogH* (*Deinococcus radiodurans* orphan glycosyl hydrolase-like family 10), which encodes a novel glycoside hydrolase, was excavated using a multi-omics combination method. The content accumulation of trehalose and its precursors under hypertonic conditions was quantified by HPLC-MS. Ours results showed that the *dogH* gene was strongly induced by sorbitol and desiccation stress in *D. radiodurans*. DogH glycoside hydrolase hydrolyzes α-1,4-glycosidic bonds by releasing maltose from starch in the regulation of soluble sugars, thereby increasing the concentration of TreS (trehalose synthase) pathway precursors and trehalose biomass. The maltose and alginate content in *D. radiodurans* amounted to 48 μg mg protein^−1^ and 45 μg mg protein^−1^, respectively, which were 9 and 28 times higher than those in *E. coli*, respectively. The accumulation of greater intracellular concentrations of osmoprotectants may be the true reason for the higher osmotic stress tolerance of *D. radiodurans*.

## 1. Introduction

Glycoside hydrolases (GHs) are enzymes that catalyze the hydrolysis of glycosidic bonds in glycoconjugates, oligo- and polysaccharides. They exist in almost all living things. Traditionally, enzymes have been grouped according to the guidelines set by the International Union of Biochemistry and Molecular Biology (IUB-MB), which is largely based on their substrate specificity [1]. Although the IUB-MB classification system is still being used, it does not reflect the structural and mechanistic features of GHs. To date, 162 glycoside hydrolase families have been described in the Carbohydrate Active Enzymes (CAZy) database, divided into 18 glycoside hydrolase GH clans (A to R) (http://www.cazy.org (accessed on 10 March 2022)). At present, the classification of GHs is mainly based on protein sequence similarity [2]. This classification scheme not only reflects the structural characteristics of GHs, but also helps to reveal the evolutionary relationships among different GHs. GHs can be divided into four broad categories: α-glucan metabolism, peptidoglycan remodeling, β-glycan hydrolysis and α-demannosylation [3]. Among these functions, α-glucan-targeting enzymes are involved in osmotic stress responses. Trehalose is a non-reducing disaccharide consisting of two glucopyranoside residues linked by an α,α-1, 1-glucoside bond. Thus far, five different trehalose synthesis pathways have been reported [4]. Most microorganisms have only one trehalose synthesis (TPS/TPP) pathway, others have two, and a few even have three or four pathways [5]. Among these pathways, the GH65 family (e.g., OtsB1 in *M. tuberculosis*) and the GH13 family (TreY maltose trehalose synthase, TreZ maltose trehalose hydrolase, TreS trehalose synthase and GlgB/GlgX glucan branching enzyme) are mainly involved in osmotic stress. The GH13 family is one of the largest families in the CAZy classification system, with more than 20 different enzyme activities reported [6].

Extremophiles are a class of microorganisms that can survive in extreme environments. They were first proposed by Elroy et al. in 1974 [7]. *Deinococcus radiodurans* can survive in extreme environments and is highly tolerant to abiotic stresses such as ionizing radiation, desiccation and oxidative stress. It is known as “the strongest microbe in the world” and has attracted much attention from scholars [8,9,10]. The genome of *D. radiodurans* was sequenced in 1999, and there are 3187 ORFs (open reading frames) in the whole genome. Of these, 1002 ORFs could not be matched in the database, and the function of these genes is still unknown [11]. According to reports, among the five natural trehalose synthesis pathways, *D. radiodurans* is reported to lack the TPP/TPS pathway and produces trehalose mainly through the TreY/TreZ (*dr0463/dr0464*) and TreS (*dr0933*) pathways [10]. The interrelation between trehalose, glycogen or maltose in vivo has been the most interesting subject in recent years [12]. The accumulation of metabolites, such as glycogen or maltose, may affect trehalose biosynthesis through the TreS and TreY/TreZ pathways [12]. On the other hand, the enhanced conversion efficiency of soluble starch to trehalose may alter trehalase activities [13,14,15]. The TreS pathway is reversible, through intramolecular glycosylation, to achieve the interconversion of trehalose and maltose in the absence of any coenzyme. How does the TreS pathway in *D. radiodurans* respond to environmental stress, which is the key enzyme and where does the precursor maltose come from? These questions deserve further investigation.

Our studies revealed an association between a glycoside hydrolase-coding gene, *dr2412,* and the environmental stress response, through comparative genomic, transcriptomic and proteomic analyses [16]. The DR2412 protein is predicted to contain a glycoside hydrolase-like family 10 (GHL10) domain, named DogH (*Deinococcus radiodurans* orphan glycosyl hydrolase-like family 10), and there is no report on the biological function of this gene at present. In this study, we explored the unique trehalose metabolism genes present in the *D. radiodurans* genome, quantified the accumulation of trehalose and its precursor metabolite maltose under hypertonic conditions and obtained insights into the trehalose synthesis pathway mediated by the novel glycoside hydrolase DogH.

## 2. Results

### 2.1. Domains of DogH and Its Bioinformatics Analysis

Multi-omics analysis showed that the transcription-level and protein-level expression of *D. radiodurans* gene *dr2412* (*dogH*) were significantly upregulated under stress treatment. It was predicted that the protein encoded by the *dogH* gene contained the GHL10 glycoside hydrolase domain.

**Figure 1:**
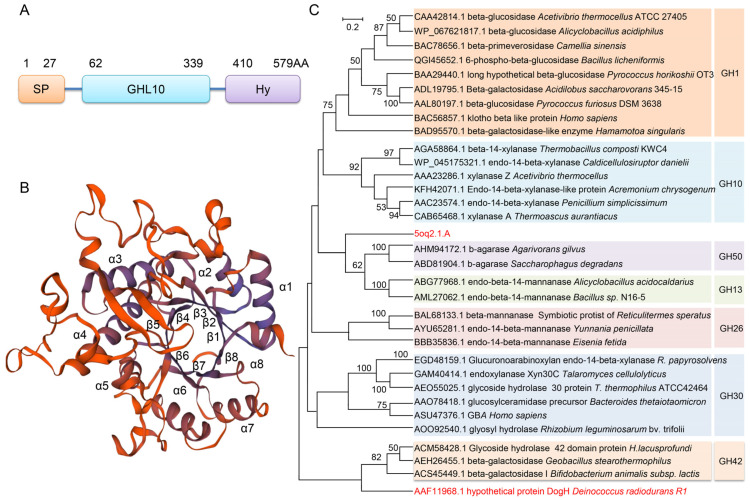
Domain architecture and phylogenetic analysis of the GHL10 domain of DogH. (**A**) Domain architecture of DogH drawn to scale. The full-length enzyme contains a predicted signal peptide (SP;residues 1 to 27), followed by a putative GHL10 domain (GHL10; residues 62 to 339) and hydrophilic end sequences (Hy;residues 410 to 579). (**B**) Ribbon diagram of the overall fold. DogH assumes a TIM barrel fold, forming an eight-stranded b-barrel surrounded by eight a-helices. The active site is formed over the center of the barrel, near the C-termini of b-strands. (**C**) Phylogenetic analysis of GH10 and other enzymes from families GH1, -10, -13, -26, -30, -42, and -50 in the GH-A clan. The tree was constructed using the maximum-likelihood method inferred by neighbor joining using 352 amino acid residues. Values above 50% are shown. Cwp19 crystal structure as a template (SMTL ID:5oq2.1.A) indicates the homologs of DogH characterized in this study.

The Uniprot database predicted that DogH was composed of 579 amino acids, including a signal peptide consisting of 27 amino acid residues, a glycosidic hydrolase GHL10 family domain (62–339 residues) and a hydrophilic terminal sequence (529–579 residues) (Figure 1A). A DELTA-BLAST search of the NCBI database showed that DogH had the highest sequence similarity (23.9% similarity, 68% coverage) to the GH10 structural domain of *Trichocoleus* sp. FACHB-591. Swiss-Model predicted the structure of DogH and showed that it had 21% sequence similarity to the Cwp19 protein from *Clostridium difficile* [17]. The Cwp19 protein contained the GHL10 domain, and we used the Cwp19 crystal structure as a template (SMTL ID: 5oq2.1.A) to simulate the folding structure of the DogH protein. DogH contains a typical glycoside hydrolase GH10 family (β/α)_8_-barrel structure, also known as the TIM barrel structure (Figure 1B). It is essentially consistent with the secondary structure predicted by PSIPRED online (Figure S1 in the Supplementary Materials).

The glycoside hydrolases of the GH-A family contain a TIM barrel structure. In this study, 32 full-length proteins from the GH-A family were selected for sequence similarity comparison, and the DogH protein was not classified into any glycoside hydrolase family (Figure 1C). The above structural features suggest that the glycoside hydrolase encoded by *dogH* does not belong to any of the resolved GH-A subfamilies and may be a novel glycoside hydrolase.

**Figure 2:**
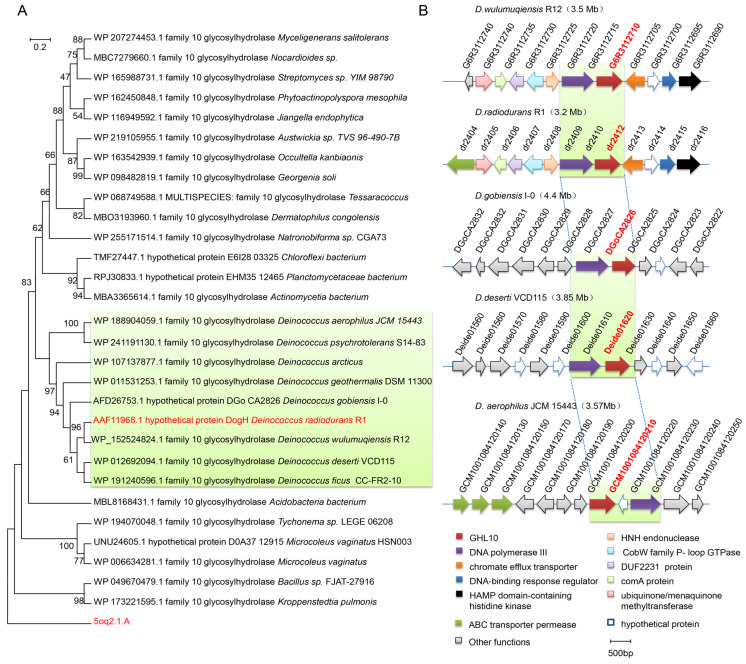
Phylogenetic analysis of DogH proteins and physical organization comparative analysis of gene clusters compared with other *Deinococcus*. (**A**) Neighbor joining tree method was used to compute the distances of DogH proteins using the MEGA 7.0 program. The *D. radiodurans* DogH protein and the 5oq2.1.A protein from uncultured bacteria are indicated with red font. (**B**) The gene organization of *dogH* homologous sequences in different *Deinococcus* species. *D. wulumuqiensis* R12; *D. radiodurans* R1; *D. gobiensis* I-0; *D.deserti* VCD115; *D. aerophilus* JCM15443. Same colors indicate similar predicted functions of the depicted ORF. Orthologous genes of *dogH* are shown in red. Orthologous genes of *dr2410* and *dr2413* are shown in purple and orange, respectively. Adjacent genes are labeled with other colors. The genome size is indicated at the end of the physical organization.

BLASTP showed that the homology between the full-length DogH and 92 putable proteins from the same genus *Deinococcus* was above 42%, and the highest similarity of 90% was obtained with *D. wulumuqiensis* R12. UniProt was searched for DogH-associated proteins from different species, none of which were experimentally characterized. The phylogenetic tree of 30 DogH-associated proteins is summarized in Figure 2A, and it suggested that DogH and the *Deinococcus* protein had high sequence similarity and were closely related. No homologous proteins were identified in any other bacterial species, with the highest sequence similarity to putative proteins from *Chloroflexi bacterium* (41.3%, with 69% coverage), but which belongs to an unclassified genus in the taxonomy. These findings suggest that the glycoside hydrolase encoded by *dogH* may belong to a protein specific to *Deinococcus*.

Comparative genomic analysis showed that a series of evolutionary events, such as gene rearrangement or gene truncation, occurred in the gene cluster composed of *dogH* and its 11 neighboring genes in other *Deinococcus* strains. All the genes were related to metabolism and environmental signal processing and were directly or indirectly involved in cellular osmotic regulation. In Figure 2B, five different strains from *Deinococcus* are listed: *D. wulumuqiensis* R12, *D. radiodurans* R1, *D. gobiensis* I-0, *D. deserti* VCD115 and *D. aerophilus* JCM15443, *dr2410* (DNA polymerase subunit III, labeled in purple) and the *dogH* gene (GHL10 glycoside hydrolase, labeled in red) were highly conserved, and *dr2411* was a pseudogene in the latest genome annotation. *D. wulumuqiensis* R12 and *D. radiodurans* R1 had high sequence similarity. They possess 97.3% similarity in the DNA-binding response regular (blue marks), 91.7% similarity in histidine kinase (black marks), 94.6% similarity in endonucrenase (light orange marks) and 87% similarity in chromate efflux transporters (orange marks), among which the two-component regulatory system has the highest similarity. *D. aerophilus* JCM 15443 and *D. radiodurans* R1 had functionally similar ABC transporter penetrases (green marks). The *Deinococcus* gene cluster results suggest that the *dogH* genes are involved in the cellular response to stress, possibly by working together with the surrounding family of membrane transport-related proteins and transcriptional regulators to coordinate their biological functions during cellular involvement in stress responses.

### 2.2. dogH Gene Deletion Reduced Osmotic and Desiccation Stress Tolerance of D. radiodurans

**Figure 3:**
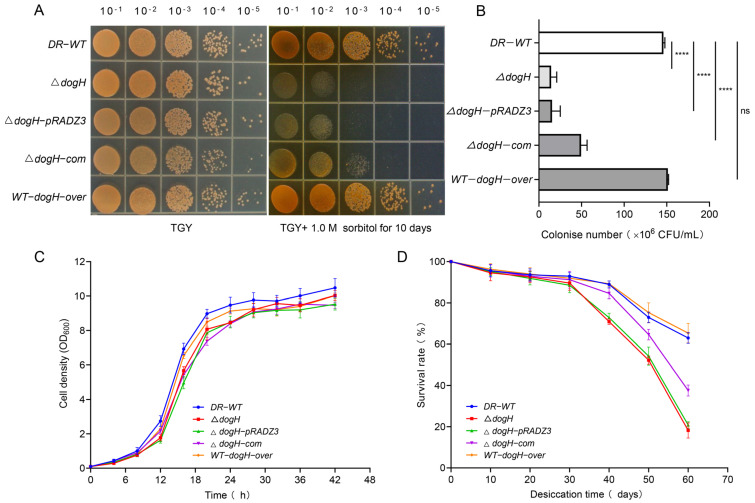
The deletion of the *dogH* gene reduced sorbitol and desiccation stress tolerance of *D. radiodurans*. (**A**) Sorbitol sensitivity assays of different *D. radiodurans* strains. Spotted agar plates after sorbitol treatment and serial dilution. (**Left**) 0 M sorbitol; (**right**) 1.0 M sorbitol for 10 days. (**B**) Statistics of colony number of different *D. radiodurans* strains under 1.0 M sorbitol stress for 10 days. (**C**) The growth curves of different *D. radiodurans* strains in TGY. (**D**) Survival curves of different *D. radiodurans* strains following exposure to desiccation. The survival rate was expressed as the percentage of the number of colonies in the treated samples compared with those in untreated controls. DR-WT: wild-type strain; Δ*dogH*; *dogH*-deleted mutant; Δ*dogH*-pRADZ3: *dogH* mutant transformed with pRADZ3 empty plasmid; Δ*dogH*-com; *dogH* mutant supplemented with the *dogH* gene; WT-*dogH*-over: wild-type overexpression with the *dogH* gene. One-way ANOVA and Dunnett’s multiple-comparison test; A probability value of *p* ≤ 0.05 was considered significant. Data are presented as averages ± SEM. ****: *p* ≤ 0.0001; and ns: non-significant.

To investigate whether the deletion of *dogH* affected the growth of the *D. radiodurans* R1 strain, this study measured the growth curves in normal culture conditions (30 °C, 220 rpm, TGY medium). The growth curves of the wild-type strain DR-WT, the deletion mutant strain Δ*dogH*, the empty plasmid backfill strain Δ*dogH*-PRADZ3, the *dogH* gene complementation strain Δ*dogH*-com and the overexpression strain WT-*dogH*-over are shown in Figure 3C. The results showed that the deletion of *dogH* did not affect the growth of the strains, and the growth status of the five strains was essentially the same, all showing a standard S-shaped curve. When the strains reached the logarithmic growth and plateau stage, the OD_600_ value of each strain did not increase.

To investigate the role of the *dogH* gene in the stress resistance process of *D. radiodurans*, the expression pattern of the *dogH* gene in response to abiotic stress was analyzed by RT-PCR first (Figure S2 in the Supplementary Materials). After 1.0 M sorbitol shock, the expression of *dogH* was upregulated by three times, indicating that the expression of the *dogH* gene was induced by osmotic stress. This was followed by a comparative analysis of the viability of *D. radiodurans* strains under abiotic stress treatment. Under different concentrations of 0.6 M, 0.8 M and 1.0 M sorbitol, compared to the wild-type strain DR-WT, the number of cell colonies of mutant Δ*dogH* decreased by three orders of magnitude under osmotic stress treatment at 1.0 M for 4 days. The results showed that the *dogH* gene was sensitive to osmotic stress (Figure S3A in the Supplementary Materials). The biological function of the gene was further elucidated by constructing a backfill strain. Compared with the wild-type strain DR-WT, the growth of the deletion mutant Δ*dogH* and the complementation control strain (Δ*dogH*-PRADZ3) was seriously affected and the number of colonies decreased by three orders of magnitude after 10 days of 1.0 M sorbitol stress. The complementation strain (Δ*dogH*-com) can partially complement the phenotype of Δ*dogH*, and the overexpression strain WT-*dogH*-over has a similar growth status to the wild-type strain DR-WT (Figure 3A,B). *D. radiodurans* cell cultures were treated in a dry environment at <5% humidity for 10, 20, 30, 40, 50 and 60 days. Survival curves for desiccation stress showed that the Δ*dogH* mutant strain decreased in growth after 60 days of desiccation by two orders of magnitude compared to the wild-type strain, and that the complementary strain Δ*dogH*-com decreased by one order of magnitude (Figure 3C,D).

Taken together, these experiments showed that *dogH* can specifically respond to osmotic stress signals and play an important role in the osmotic stress response.

### 2.3. dogH Gene Deletion Affected the Intracellular Osmoprotectant Content of D. radiodurans

**Figure 4:**
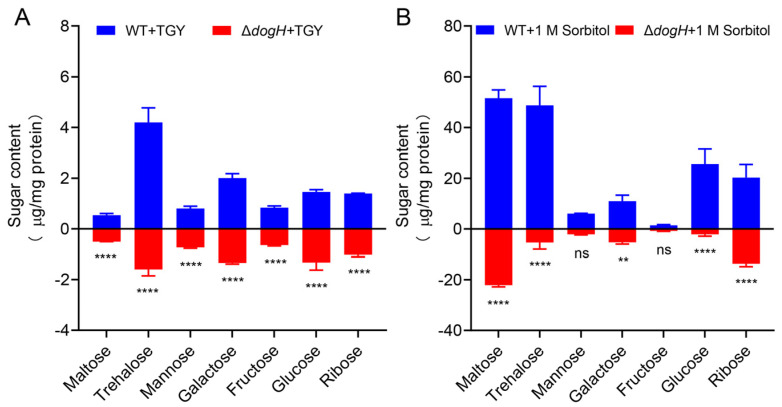
GC-MS quantitative analysis of carbohydrate metabolites in *D. radiodurans*. (**A**) Without sorbitol treatment; (**B**) with 1.0 M sorbitol stress treatment for 3 h. WT: wild-type strain; Δ*dogH*: *dogH*-deleted mutant. One-way ANOVA and Dunnett’s multiple-comparison test; A probability value of *p* ≤ 0.05 was considered significant. Data are presented as averages ± SEM. ****: *p* ≤ 0.0001; **: *p* ≤ 0.01; and ns: non-significant.

The *dogH* gene in *D. radiodurans* encodes a genus-specific glycoside hydrolase. To elucidate the biological functions of *dogH*, 24 types of intracellular small-molecule carbohydrate metabolites were screened and determined by the GC-MS method (Table S1 in the Supplementary Materials). Under normal growth conditions (Figure 4A), seven small molecules of sugars were slightly different in the wild-type strain compared to the mutant strain Δ*dogH*. The maltose and trehalose content in the wild type were approximately 1.4 times and 2.6 times higher than those in ∆*dogH*, respectively. As shown in Figure 4B, the metabolites of the *D. radiodurans* wild-type and mutant strain Δ*dogH* differed significantly under 1.0 M sorbitol stress treatment for 3 h (Figure S4 in the Supplementary Materials). Seven soluble sugars, i.e., maltose, trehalose, mannose, galactose, fructose, glucose and ribose, were able to accumulate in large amounts under stress, with the most significant differences in maltose, trehalose and glucose. The maltose and trehalose content in the wild-type were 56.6 μg mg protein^−1^ and 48.7 μg mg protein^−1^, respectively. These were approximately 2.6 times and 9.2 times higher than those in Δ*dogH* under osmotic stress.

These data indicate that the DogH glycoside hydrolase may counteract osmotic stress by hydrolyzing certain carbohydrates in the cell to produce osmoprotectants, agents to balance osmotic stress.

Meanwhile, in this study, the transcriptional expression of related genes in the synthesis pathway of maltose and trehalose was evaluated by RT-PCR under 1.0 M sorbitol stress shock for 3 h. The results showed that the expression of related genes in maltose and trehalose synthesis pathway could be induced by osmotic stress, and the upregulation ratio ranged from 3 to 10 times, among which *dogH* was upregulated by 2.8 times, as shown in Table 1. These results indicate that the upregulated expression of genes in the synthesis pathway of maltose and trehalose induces a coordinated cell response to osmotic stress.

### 2.4. DogH Glycoside Hydrolase Substrate Specificity and Product Analysis

The DogH protein was isolated and purified from *E. coli* BL21 (DE3) in order to analyze the substrate specificity of the DogH glycoside hydrolase (Figure S5 in the Supplementary Materials). In this study, nine carbohydrates containing different glycosidic bonds were selected to assess substrate specificity, as listed in Table 2. The final concentrations of reducing and non-reducing sugars were determined to be 0.25% and 0.5%, respectively (Figure S6 in the Supplementary Materials), with reference to the method shown in Section 4.7.

**Figure 5:**
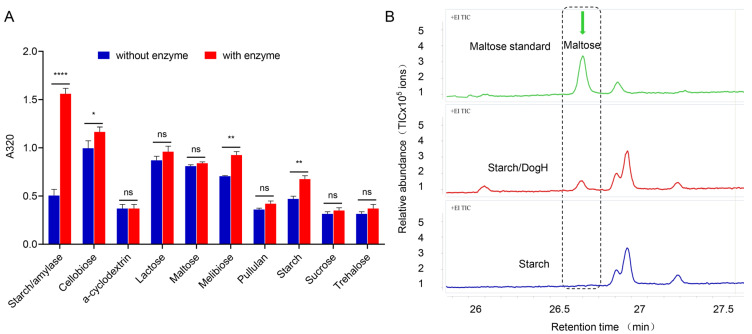
Substrate specificity and product analysis of DogH glycoside hydrolase. (**A**) Detection of carbohydrate breakdown. A significant difference was observed in starch/amylase control; some differences in DogH in the carbohydrate test were significant, such as cellobiose, melibiose and starch. (**B**) Quantitative determination of decomposition products of DogH glycoside hydrolase by HPLC-MS. Green peak; maltose standard; red peak: addition of DogH glucoside hydrolase to starch to produce maltose products; blue peak: no DogH glycoside hydrolase is added to starch, so maltose is not produced. One-way ANOVA and Dunnett’s multiple-comparison test; A probability value of *p* ≤ 0.05 was considered significant. Data are presented as averages ± SEM. ****: *p* ≤ 0.0001; **: *p* ≤ 0.01; *: *p* ≤ 0.05; and ns: non-significant.

Starch was hydrolyzed by amylase as a positive control, by the addition of DogH as an experimental group and without DogH as a negative control group. As shown in Figure 5A, DogH had no significant catalytic activity against α-cyclodextrin, lactose, maltose, pullulan, sucrose and trehalose, indicating that this glycoside hydrolase could not cleave the β-1,4, α-β-1,2, α-α-1,1 and other glycosidic bonds. Notably, DogH can hydrolyze three substrates, indicating that DogH has high catalytic cleavage efficiency for cellobiose, melibiose and starch containing a β-glucosidic bond, α-1,6 and both α-1,4 and α-1,6 glycosidic bonds, respectively.

According to the differential expression analysis of seven types of osmoprotectants in the cells of *D. radiodurans* determined by GC-MS, as shown in Figure 4A,B, it was speculated that the substrates of the DogH glycoside hydrolase might exist in seven types of sugar synthesis pathways. Therefore, the potential substrates containing α-/β-1,4 and α-1,6 glycosidic bonds were screened. Among the seven sugar synthesis pathways of maltose, trehalose, mannose, galactose, fructose, glucose and ribose, only starch (α-1,4/α-1,6) and mannan (α-1,6) could be specifically hydrolyzed by the DogH glycoside hydrolase. The hydrolysis products of the DogH protein were analyzed by HPLC-MS, using starch and mannose as substrates. As shown in Figure 5B, the maltose standard product has a single sharp peak (26.606 min, green peak plot). Compared with the maltose standard product, starch catalyzed by the DogH glycoside hydrolase can produce a certain amount of maltose (26.603 min, red peak plot), while maltose cannot be detected in the product without DogH (blue peak plot). No mannose hydrolysate was detected in this experiment.

These findings suggest that DogH glycoside hydrolase can hydrolyze starch to produce maltose and accumulate a greater substrate concentration for the TreS pathway in *D. radiodurans*. It is speculated that this reaction may further affect the trehalose biomass and thus enhance cellular osmotic tolerance.

### 2.5. DogH Affected Trehalose Content of D. radiodurans

**Figure 6:**
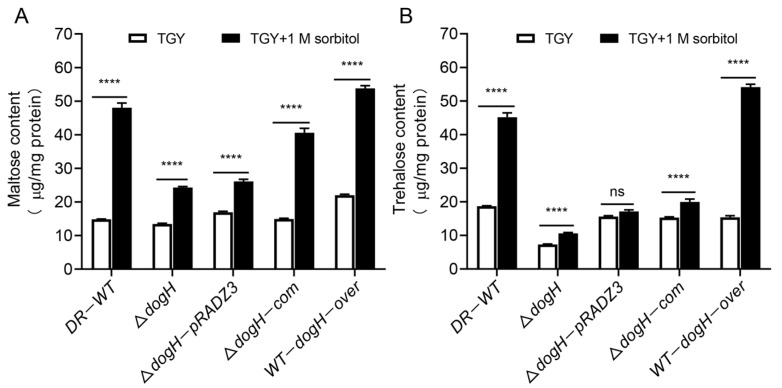
Determination of osmoprotectant content in different *D. radiodurans* strains by HPLC-MS. (**A**) Maltose content. (**B**) Trehalose content. DR-WT: wild-type strain; Δ*dogH*:*dogH*-deleted mutant; Δ*dogH*-pRADZ3: *dogH* mutant transformed with pRADZ3 empty plasmid; Δ*dogH*-com: *dogH* mutant supplemented with the *dogH* gene. WT-*dogH*-over: wild-type overexpression with the *dogH* gene. 1.0 M sorbitol stress treatment for 3 h treated (black bars) and nontreated (white bars). One-way ANOVA and Dunnett’s multiple-comparison test; A probability value of *p* ≤ 0.05 was considered significant. Data are presented as averages ± SEM. ****: *p* ≤ 0.0001; and ns: non-significant.

To further demonstrate the relevance of *dogH* for TreS-mediated trehalose content in *D. radiodurans*, the maltose and trehalose of different strains of *D. radiodurans* were quantitatively determined by HPLC-MS. As shown in Figure 6A,B, under normal growth conditions, maltose and trehalose content were slightly reduced in the Δ*dogH* mutant strain, Δ*dogH*-pRADZ3 supplement empty plasmid strain and Δ*dogH*-com complementary strain compared to the wild-type DR-WT, and trehalose was decreased by 2.6 times to 7.2 μg mg protein^−1^. The maltose and trehalose content in the WT-*dogH*-over overexpression strain were comparable to those in the wild-type DR-WT.

Under 1.0 M sorbitol stress for 3 h, maltose and trehalose content were significantly increased compared to normal growth conditions, and the results were consistent with those shown in Figure 4A,B. Compared with the Δ*dogH* mutant, the content of maltose in the Δ*dogH*-com complementary strain increased from 24.2 μg mg protein^−1^ to 40.6 μg mg protein^−1^, an increase of approximately 1.7 times, and the increase in trehalose, from 10.6 μg mg protein^−1^ to 20 μg mg protein^−1^ amounted to two times. The results indicated that the *dogH* gene could affect the content of maltose and trehalose.

Taken together, these experiments showed that the DogH glycoside hydrolase could produce maltose by hydrolyzing starch and increasing the concentration of maltose in the substrate in the TreS pathway, thus further regulating the changes in trehalose content in *D. radiodurans* R1 and improving the cellular osmotic stress tolerance.

### 2.6. Different Environments Shape Strains with Different Osmoprotectants

**Figure 7:**
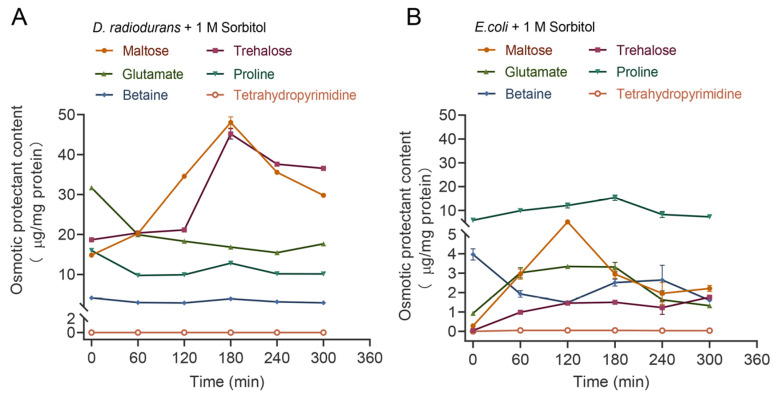
Composition of the compatible solute pool of different strains under 1.0 M sorbitol stress. (**A**) *D. radiodurans* R1 wild-type. (**B**) *E. coli* DH5α. Filled circles, maltose; filled squares, trehalose; filled triangles, glutamate; filled inverted triangles, proline; filled diamonds, betaine; open circles, tetrahydropyrimidine.

As a Gram-positive bacterium, *D. radiodurans* can survive under extreme conditions of drought, radiation and nutrient deprivation. *E. coli*, as a Gram-negative bacterium, can tolerate different osmotic pressures in the anaerobic gastrointestinal tracts of mammals. In order to avoid the adverse damage caused by hypertonic stress, cells accumulate osmoprotectants in the cytoplasm through synthesis or ingestion. In this study, HPLC-MS was used for the quantitative analysis of the accumulation of various osmoprotectants in *D. radiodurans* and *E. coli* strains after 300 min of treatment with 1.0 M sorbitol stress (Figure 7).

In the wild-type strain of *D. radiodurans*, the intracellular tetrahydropyrimidine content was extremely low and did not change significantly with increasing stress time, indicating that it may not be involved in the long-term adaptive response of *D. radiodurans* to osmotic stress. Proline, glutamic acid and betaine content showed a very sharp decrease after 60 min stress treatment, followed by stable content with no significant difference. In contrast, HPLC-MS clearly detected significant changes in intracellular carbohydrate concentrations, showing an increasing trend followed by a decreasing trend. The trehalose concentration increased from 18 μg mg protein^−1^ to 45 μg mg protein^−1^ under high osmotic pressure for 180 min, indicating an increase by approximately 2.5 times. Maltose content was observed to have increased by approximately three times, from 15 μg mg protein^−1^ to 48 μg mg protein^−1^ (Figure 7A). The above experiments suggest that sugar solutes are the main osmoprotectant agents in *D. radiodurans*.

In the wild-type strain of *E. coli* DH5α, the intracellular tetrahydropyrimidine content was similar to the accumulated phenotype of *D. radiodurans*. In contrast, other solutes changed significantly, with proline concentrations increased to 15.4 μg mg protein^−1^ by 180 min of treatment, an approximately three times increase. Glutamic acid content was observed to have increased by around three times (from 1 μg mg protein^−1^ to 3.3 μg mg protein^−1^). Trehalose content gradually increased with time, increasing to 1.6 μg mg protein^−1^ at 300 min. Maltose content increased by approximately 17 times to 5.2 μg mg protein^−1^ by 120 min (Figure 7B). The above experiments indicate that amino acid solutes are the main osmoprotectant agents in *E. coli*.

Analysis of the osmoprotectants showed that *D. radiodurans* accumulated more trehalose and maltose than *E. coli*, which may confer higher tolerance to osmotic stress in *D. radiodurans*.

## 3. Discussion

### 3.1. The Evolutionary Origin of DogH Glycoside Hydrolase

The extreme-radiation-tolerant bacterium *D. radiodurans* is closely related evolutionarily to the extremely thermophilic bacterium *Thermus thermophilus* and is considered to belong to a distinct branch of bacteria known as the *Deinococcus-Thermus* group [18]. After radiation from a common ancestor, the two genera underwent different paths towards their different lifestyles. The main evolutionary processes leading to this genomic divergence included (i) differential gene loss and acquisition. (ii) gene acquisition through horizontal gene transfer (HGT) followed by the possible loss of lineal homologous genes. *D. radiodurans* obtained genes from different bacteria through HGT that accounted for 10–15% of the genome (such as genes involved in inorganic ion transport and metabolism), and these genes significantly enhanced its ability to survive under different types of environmental pressure [19,20]. (iii) Genealogy-specific expansion through HGT replication and/or the acquisition of paralogous homologous gene families also occurred [21], i.e., *D. radiodurans* membrane-associated protein family (DR2080, DR1043, DR1952, DR1953, DR1738) and its adjacent PadR family transcriptional regulatory factors (COG1695;9 paralogs in DR), which together participate in the regulation of cell stress to chemical reactions [22]. (iv) protein stability may be affected by amino acid composition modifications.

Based on the sporadic distribution of *dogH* in bacteria and the highly conserved subgene clusters, we speculated that horizontal gene transfer was possible in *Deinococcus*. In contrast to host-specific pathogens and symbionts with decreasing genomes, *Deinococcus* continuously expand their genomes through gene transfer events, resulting in highly adaptive extremophiles that contribute to their extreme tolerance [23,24]. In addition, NCBI prediction analysis showed that the DogH protein contains a GHL10 structural domain. Compared with the domains derived from plants, bacteria and archaea, the GH10 domain of the DogH protein has higher similarity (40.7–91.2%) to *Deinococcus*, but the consistency with other sources of the GH10 domain is less than 25%. This suggests that the GH10 domain may have different biological functions in different species. In this case, the inconsistent distribution of GH10 structural domains may be a result of the selective retention of defence mechanisms during evolution. Signal 5.0 predicts that the DogH protein contains a 27 amino-acid signal peptide, and our analysis of the nine *Deinococcus* DogH proteins, seen in Figure 2A, showed that eight congenic proteins contained signal peptide, except for *D. psychrotolerans* S14-83. Therefore, it can be speculated that the DogH protein coordinates its biological functions by targeting the cytoplasmic membrane in the process of the stress response to form osmotic-stress-related gene clusters. This gene cluster consists of DogH proteins and the surrounding membrane-associated protein family, as well as transcriptional regulators specifically amplified from the parageneic family of genes.

Expasy predicts that *dr2410* (DNA polymerase III), an adjacent gene to the *dogH* gene (*dr2412* gene), contains a disordered hydrophilic fragment in the C-terminal, which belonged to a hydrophilic protein. This protein belongs to the AAA+ superfamily of ATPases, which are present in all organisms, and they are involved in a variety of cellular processes, including membrane fusion, proteolysis and DNA replication. Recent structural studies have revealed that they usually form ring-shaped oligomers, which are crucial for their ATPase activities and mechanisms of action. These ring-shaped oligomeric complexes are versatile in their modes of action, which collectively seem to involve some form of disruption of molecular or macromolecular structure, the unfolding of proteins, the disassembly of protein complexes, the unwinding of DNA or the alteration of the state of DNA protein complexes. Thus, the AAA+ proteins represent a novel type of molecular chaperone that may play a role in the resistance of DogH proteins to adversity stress [25,26].

**Figure 8:**
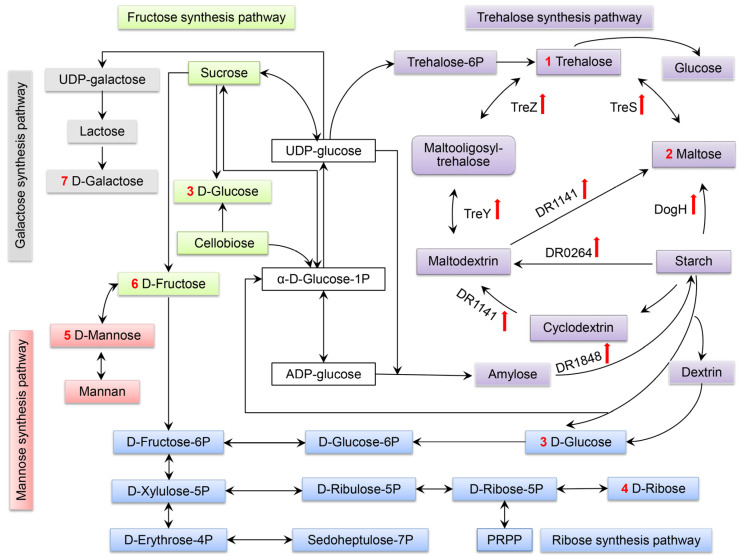
Regulation diagram of carbohydrate osmoregulation pathway. Note: purple pathway: glucose, trehalose and maltose; blue pathway: glucose and ribose; pink pathway: mannose; green pathway: fructose; grey pathway: galactose. The genes and numbers in the remarks indicate the RT-PCR expression amount of related genes in the TreY/TreZ and TreS pathways, under the condition of 1.0 M sorbitol stress treatment for 3 h.

Redundancy is a distinctive feature of *D. radiodurans* [10]. It contains two amylases, DR1472 (function unknown, presumably containing α-amylase catalytic structural domain) and DR1141. In the starch hydrolysis pathway, gene *dr1141* is predicted to encode cyclomaltodextrinase/maltogenic alpha-amylase/neopullulanase, which may degrade starch into maltodextrin or maltose in response to sorbitol stress. The expression level increased by 3.9 times, as shown in Figure 8. DogH belongs to the GH-A clan and uses a retention mechanism to act at the catalytic site containing two glutamate residues [27]. DR1141 belongs to family 13 of the GH-H clan and the catalytic nucleophilic base is aspartate. Whether DR1141 and DogH have synergistic effects in the starch metabolic pathway will be investigated in our next work.

### 3.2. Pathways of Trehalose Synthesis in D. radiodurans

Trehalose is generally believed to play a role in the protection of cells and biomolecules by replacing hydrogen bonds with molecularly bound water, or by participating in the formation of a glassy matrix in the cytoplasm. The above studies demonstrate that different strains produce different osmoprotectants. This may be because under different osmotic stress conditions, the preference of osmoprotectants is related to the stable protection degree of macromolecules such as enzymes [28]. Alternatively, different cellular responses to osmotic stress may result from the ability to accumulate trehalose and other osmoprotective agents. This ability ultimately depends on the presence of the genes for their respective biosynthetic pathways [29].

Trehalose is a disaccharide accumulated by many microorganisms under stress conditions. It contains an α,α-1, 1-glycosidic bond and is known for its strong osmotic protection, strong stability and low reactivity. It serves as a source of energy and carbon, a bacterial cell wall component and a signalling molecule. It is considered to be a universal stress molecule that protects cells and biomolecules from injuries imposed by high osmolarity, heat, oxidation, desiccation and freezing. It is also a free radical scavenger [30]. To date, five different pathways of trehalose synthesis have been reported. (i) The TPS/TPP pathway is the most common, performing the transfer of glucose from an NDP-glucose donor to glucose-6-phosphate and subsequent dephosphorylation to produce trehalose. It mainly involves two enzymes: trehalose 6-phosphate synthase (TPS) and trehalose-6-phosphate phosphatase (TPP). This pathway exists in all prokaryotes and eukaryotes that synthesize trehalose [12,31,32,33]. In *E. coli*, this pathway was named the OtsA/OtsB pathway. (ii) Trehalose can also be isomerized from the α1-α4 bond of maltose to the α1-α1 bond of trehalose by *treS*-encoded trehalose synthase (TreS pathway), which has been described in the mycobacterium *Mycobacterium* sp. [29,34]. (iii) A third pathway found in several bacteria converts the terminal unit of a glucose polymer to trehalose via maltooligosyl trehalose synthase, encoded by *treY*, and maltooligosyl trehalose trehalohydrolase, encoded by *treZ* [35,36]. (iv) Trehalose phosphorylase (TreP) catalyzes the synthesis of trehalose-1-phosphate and glucose, a pathway that has only been demonstrated in in vitro experiments with fungi [37]. (v) The final, less common pathway involves the conversion of ADP-glucose and glucose to trehalose via trehalose glycosyltransferring synthase (TreT), and has been characterized in hyperthermophilic archaea *Termococcus litoralis* and *Pyrococcus horikoshii* [38,39]. Most microorganisms have only one trehalose synthetic pathway, (TPS/TPP), others have two, and a few even have three or four pathways [5].

*D. radiodurans* R1 lacks the TPS/TPP pathway and produces trehalose mainly through the TreY/TreZ and TreS pathways [10]. In *E. coli*, trehalose is mainly synthesized through the OtsA/OtsB pathway [12]. It has been proposed that among the three pathways found in the mesophilic bacterium *Corynebacterium glutamicum*, the TreY/TreZ pathway could be involved in osmoadaptation; the TreS pathway in trehalose catabolism; and the TPS/TPP pathway would provide unknown regulation [4]. Interestingly, in *T. thermophilus* RQ-1, the TPS/TPP pathway is involved in osmoadaptation, while the function of TreS is not yet known [40]. To further confirm whether the TreY/TreZ- and TreS-mediated changes in trehalose synthesis were based on osmotically responsive expression regulation, the transcript levels of the relevant genes in the trehalose synthesis pathway were determined by RT-PCR under 1.0 M sorbitol stress shock for 3 h. The results showed that related genes in the TreY/TreZ and TreS pathways could all respond to osmotic stress, as shown in Figure 8 and Table 1. Among the two trehalose synthesis pathways, the question of which pathway plays a major role or is synergistically mediated will be further investigated in subsequent studies by constructing single- and double- mutant strains. The above results suggest that the upregulated expression of TreY/TreZ and TreS pathway genes ensures a coordinated cellular response to osmotic stress shocks.

The lifestyle and metabolism of *D. radiodurans* suggests that this bacterium is a food scavenger for the production (or composition) of other (dead) organisms: it feeds on the degradation of amino acids in proteins and sugars in polymers by secreting various hydrolytic enzymes [41]. DogH’s signal peptide contains 27 amino acids, which may play a hydrolytic role around the cell membrane. We speculate that DogH may produce maltose after the degradation of starch in the extracellular domain, and then transport maltose into the intracellular domain by the maltose transport system osmotic enzyme MalF/MalG (*dr0562*/*dr0563*), thus mediating the TreS pathway.

### 3.3. Mechanisms of Osmoregulation in D. radiodurans

**Figure 9:**
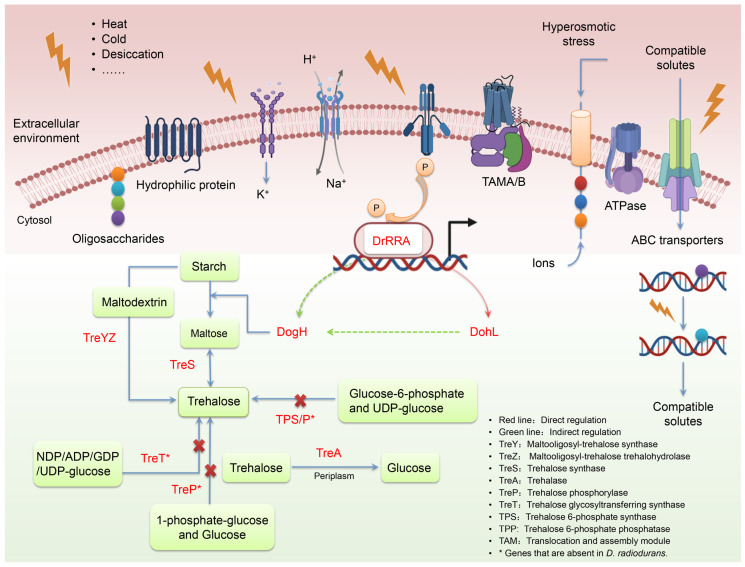
Overview of the osmoregulation mechanisms developed by *D. radiodurans*. Note: Ionic balance is maintained by transporting excessive ions out of the cell through ABC transporters; compatible solutes are accumulated to maintain the cytoplasmic activity of cells by either internal synthesis or transport from the surroundings through secondary carriers and ABC-type transporters, inducing hydrophilic protein expression to resist stressful situations.

Osmotic stress, caused by abiotic stresses such as desiccation, salt stress, high temperatures and freezing, is one of the main stresses suffered by microorganisms. In order to adapt to environmental stress, microorganisms have evolved a series of mechanisms that aid in sensing, responding to, resisting and regulating abiotic stress, namely osmotic regulation mechanisms [42]. Understanding how bacteria living in extreme environments adjust their metabolic activity and increase the resistance of microorganisms to stress is important for the optimization of biological processes [43]. The exposure of cells to desiccation, high-concentration solutes and other environments can easily lead to cell dehydration, which is eventually manifested as osmotic stress. Osmotic stress results in slow microbial growth by inhibiting genes related to metabolism, cell wall biogenesis, DNA and protein synthesis [44]. Dehydration-tolerant organisms have different means of protecting their cellular macromolecules from the harmful effects of water deprivation. In Figure 9, the regulatory mechanism of *D. radiodurans’* tolerance to osmotic stress is summarized. From potassium accumulation to protein regulation, these mechanisms play a synergistic role in cellular osmosis regulation.

The maintenance of ion homeostasis is a major protective mechanism for microbial cells against hyperosmotic stress. Ion transport channel proteins DR0927, DR0957 and DR1012, or peptide channel protein DR2155 in *D. radiodurans*, participate in ion homeostasis by transporting excess ions out of the cell. Recent studies have shown that there are KdpB polypeptides with unique tissue characteristics in *D. radiodurans*, and kdp operons can be rapidly expressed only when cells experience K^+^ restriction, and not when osmotic pressure increases [45]. The *D. radiodurans* genome contains a *tamB* homologous gene (*dr146T*). *dr146T* is involved in maintaining cell envelope integrity, and the mutation of *dr146T* leads to cell envelope spallation and reduced shear stress and osmotic resistance [46]. Hydrophilic proteins include late embryonic development-rich (LEA) proteins such as DrWhy and DohL [47]; such proteins can accumulate in large quantities in cells following desiccation or high osmotic stress. Several functions have been proposed for these proteins: molecular chaperones, hydration buffers, membrane stabilizers and ion receivers [48,49]. Several other proteins with disordered hydrophilic fragments have recently been identified in *D. radiodurans*, including DNA polymerase III, Nudex hydrolase DR0550 and ABC transporter protein DR2145 [10,50]. Their hydrophilic tails may increase the likelihood that these proteins will remain solubilized while others are denatured due to dehydration.

Different environmental strains shape different osmoprotectants. We have a fairly complete picture of the distribution of osmoprotectants in most groups of halophilic bacteria and archaea, which, in some cases, is correlated with their position in the phylogenetic tree of life [51]. Trehalose and glutamate synthesis, or the accumulation of glycine betaine upon transport from the external medium or choline oxidation, are far more widespread in the environment [5]. The *D. radiodurans* genome lacks a gene for the de novo synthesis of the osmoprotectant betaine GBase, but it has genes encoding transport proteins of GB, trehalose and phosphate ions [44]. The genomes of the thermophilic bacterium *T. thermophilus* [52] and the hyperthermophilic archaea *Pyrococcus horikoshii* and *Thermococcus* [53] would accumulate trehalose under osmotic stress. This could explain the differences in response to high osmolarity in different strains, one being able to synthesize osmoprotectants while the other needs to internalize them from the environment.

Transcriptional regulators play a key role in the regulation of gene expression. Thus far, some important transcriptional regulators, e.g., DdrO, IrrE, PprM, OxyR, IrrI, Mur, Sig and DrRRA, have been identified in *D. radiodurans* R1 [54]. The two-component system is a universal stress-resistance mechanism through which bacteria can sense and respond to a wide range of signals and stressors. *D. radiodurans* contains 23 histidine kinases and 29 response regulators in collaboration with them, and DrRRA (DR2418) is identified as a response regulator that plays a central role in the extreme resistance of *D. radiodurans* [55]. Previous studies in our laboratory have shown that *dohL* gene expression is regulated by DrRRA proteins, which encode the LEA3 family of molecular chaperone proteins that protect enzyme activity and stabilize enzyme structures under conditions of stress, such as oxidative and desiccation stress. We hypothesize that the DohL protein also protects the DogH glycoside hydrolase to some extent from folding correctly under osmotic stress, forming a complex with the target molecule to prevent the aggregation of the target molecule and thus maintaining its stable enzymatic activity properties [55]. The *dogH* gene promoter region is predicted to contain a DrRRA transcriptional regulator binding site (CTCCGGCAGCGGACCCGGTGCCTGCTC), but there is no direct experimental evidence for this. In the next step stage of our research, we will focus on the regulatory mechanisms specific to *D. radiodurans* associated with *dogH*.

## 4. Materials and Methods

### 4.1. Strains and Plasmids

The liquid media used were TGY (1% tryptone, 0.1% glucose, 0.5% yeast extract, pH 7.0) and LB (1% tryptone, 1% yeast extract, 0.5% sodium chloride, pH 7.0). Solid medium was added to TGY and LB liquid with 1.5% agar. *D. radiodurans* R1 and its recombinant bacteria were incubated in TGY at 30 °C or on TGY with appropriate antibiotics. *E. coli* was grown at 37 °C in LB or on LB using appropriate antibiotics. All liquid cultures were cultured in a shaker at 220 rpm. The plasmids and strains used in this study are listed in Table 3.

Construction of *dogH* deletion mutants and complementary strains. The mutant strains were constructed according to the principle of homologous recombination, with primers designed according to the full sequence of the *dogH* gene; see Table S2 in the Supplementary Materials. Using the *D. radiodurans* R1 genome as a template, P1/P2 amplified the upstream fragment of *dogH* at 595 bp (U) and P5/P6 amplified the downstream fragment of *dogH* at 577 bp (D). Using the pKatAPH3 plasmid as a template, P3/P4 amplified the kanamycin resistance gene *nptII* fragment 970bp (K). A homologous recombinase was used to catalyze the fusion of the three fragments, UKD, in a 1:1:1 molar ratio. P1/P6 primers were used as templates to amplify the fusion fragment UKD. Finally, the fusion fragments were imported into *D. radiodurans* R1 according to the experimental method previously reported [56]. Single colonies were screened for kanamycin resistance (20 μg/mL). The deletion of the *dogH* gene in the mutant was confirmed by PCR and DNA sequencing; see Figure S7 in the Supplementary Materials. The results confirmed that the *nptII* gene was inserted in the correct position, replacing the *dogH* gene. The obtained *dogH* deletion mutant was named Δ*dogH* and used for further studies. The *dogH* gene was inserted between the nucleic acid endonuclease *BamH* I and *Spe* I sites of the pRADZ3 plasmid, and the recombinant plasmid was then transformed into the Δ*dogH* mutant strain and wild-type *D. radiodurans* R1 for the construction of the complementary strain ∆*dogH*-com and the overexpression strain WT-*dogH*-over.

### 4.2. Bioinformatics Analysis

Using the SignalP 5.0 software [57], we performed an analysis of DogH sequence signal peptides in *D. radiodurans* R1 strains and searched for homologous GH10 structural domains according to the UniProt database. The GH10 sequence was downloaded from the UniProt database and the glycoside hydrolase database CAZy, and the DogH sequence similarity was assessed using the BLASTp algorithm, while multiple sequence alignment was performed in MEGA 7.0 [58]. Sequences that did not contain conserved catalytic residues were removed from the alignment and the neighbor joining method was applied [59]. The results of the CLUSTAL X alignment were used to construct a phylogenetic tree using the MEGA 7.0 software. Two sets of evolutionary trees containing 33 sequences of glycoside hydrolase and 30 sequences of DogH protein were selected for analysis. We used SWISS-MODEL [60] to construct structural models based on the closest PDB homologs (crystal: 5oq2.1.A, Cwp19 protein [17]) and visualized the predicted structure in Adobe Photoshop. We then used the SWISS-MODEL server to identify and align putative homologs of the GHL10 domain (residues 62 to 339) of unknown function.

### 4.3. Abiotic Stress Phenotyping

Osmotic stress tolerance assay. A 1 mL sample of *D. radiodurans* R1 and its derivatives (with appropriate antibiotics) was incubated in TGY medium until the early stages of logarithmic growth (OD_600_ = 0.6~0.8) and serially diluted 10 times with phosphate buffer to a final concentration of 10^−1^~10^−5^. Referring to the reported method [61], the growth phenotypes were recorded by incubating the samples on the surface of TGY solid medium with different concentrations of sorbitol at 30 °C for several days.

Desiccation resistance assay. A 1 mL sample of *D. radiodurans* R1 and its derivatives (with appropriate antibiotics) was incubated in TGY medium until the early stages of stable growth (OD_600_ = 8~10)and stored in a desiccator at room temperature for 60 days at relative humidity of less than 5%, following the reported method [49]. It was stored at room temperature in a desiccator at less than 5% relative humidity for 60 days. Samples were taken at 10-day intervals for cell recovery and diluted to a final concentration of 10^−1^~10^−5^. An appropriate amount of the diluted sample was dropped onto the surface of the TGY solid medium and incubated in an incubator at 30 °C for several days to record the growth phenotype.

Survival is expressed as a percentage of the number of colonies in treated samples versus untreated control samples.

### 4.4. Real-Time Fluorescence Quantitative PCR

*D. radiodurans* R1 organisms were collected under various abiotic stress conditions using the ambion^®^ RNA extraction kit from Life. Referring to the reported method [62], 16S was used as the internal reference gene, and a real-time quantitative polymerase chain reaction (qRT-PCR) was performed for *dogH*, *dr0264*, *dr1141*, *dr1848*, *treY*, *treZ* and *treS* genes under different experimental conditions via the ^△△^Ct method. The relevant primers involved in this experiment are shown in Supplementary Table S2 and the qRT-PCR results are shown in Table 1.

### 4.5. Collection and Analysis of Cellular Metabolites

A sample volume of 20 mL of *D. radiodurans* R1 and its derivatives (with the addition of the appropriate antibiotics) was collected and cultured in TGY medium until the early stages of logarithmic growth (OD_600_ = 0.6~0.8), treated for 0–5 h under 1.0 M sorbitol stress, washed twice repeatedly with ddH_2_O and finally resuspended with 3 mL of ddH_2_O. The cells were then centrifuged at 4 °C for 20 min at 12,000× *g*. The supernatant obtained was used for metabolite content analysis.

GC-MS quantitative analysis: 100 μL of sample and 400 μL of ddH_2_O were mixed and added to a 1.5 mL loading vial and pre-frozen overnight at −80 °C. The samples were lyophilized and derivatized the following day (derivatization conditions: 500 μL pyridine and 500 μL BSYTA derivatization reagent, mixed by shock and incubated in metal bath at 70 °C for 90 min). Finally, quantitative GC-MS analysis was performed. The mixture was analyzed in full scan acquisition mode using a gas chromatograph-triple quadrupole mass spectrometer (Agilent 7890A/7000 GC-QQQ) equipped with an electrospray ionization source and in separation mode using a gas chromatographic column HP-5MS 5% phenyl Methyl Silox (325 °C: 30 m × 250 μm × 0.25 μm, Agilent, Santa Clara, CA, USA). The binary isocratic separation of the water-soluble fraction containing the permeate protectant was performed using a mobile phase of 25% Milli-Q pure water and 75% pure acetonitrile (*v*/*v*%) at a flow rate of 1 mL/min, an injection volume of 10 μL and a detection time of 45 min. The initial column chamber temperature was increased from 100 °C to 150 °C (holding time 2.5 min) and finally to 310 °C (holding time 44.5 min). The samples were run according to the above conditions and the retention times and mass spectral peak plots were compared and analyzed against standards; the types of standard components used for calibration are shown in Supplementary Table S1, purchased from Sigma (St. Louis, MO, USA). Meanwhile, the total protein content of the supernatant was determined using the Komas Brilliant Blue assay [63]. The number of micrograms of metabolite per mg of protein in the supernatant was calculated and the solute concentration was expressed as µg mg protein^−1^. Results of the GC-MS assay are shown in Supplementary Figure S4.

Quantification of sugars and other substances by HPLC-MS: 500 μL of sample and 500 μL of methanol extraction were mixed and added to a 1.5 mL loading vial for the quantification of cellular metabolites by HPLC-MS. A binary gradient procedure consisting of mobile phase A pure water (0.3% ammonia) and mobile phase B pure acetonitrile was used to achieve the chromatographic separation of compatible substances: 0–7 min: 15% of phase A and 85% of phase B; 7 min: 40% of phase A and 60% of phase B; 7.1–10 min: 15% of phase A and 85% of phase B. The above injection volumes were fed according to volume fraction (*v*/*v*%) percentages. The sample injection time was 10 min, injection volume 10 μL, flow rate 0.3 mL/min, column temperature 100 °C.

Quantification of amino acids by HPLC-MS: 500 μL of sample and 500 μL of methanol extractant were mixed and added to a 1.5 mL loading vial for the quantification of cellular metabolites by HPLC-MS. A binary gradient procedure consisting of mobile phase A pure water (20 mM ammonium acetate) and mobile phase B pure acetonitrile, pH 3.0, was used to achieve the chromatographic separation of compatible substances: 0 min: 0% phase A, 100% phase B; 0–11.5 min: 30% phase A, 70% phase B; 12–15 min: 0% phase A, 100% phase B. The above injection volumes were based on volume fraction (*v/v*%) percentage injection. The sample injection time was 15 min, injection volume 10 μL, flow rate 0.5 mL/min, column temperature 100 °C.

A liquid chromatograph triple quadrupole mass spectrometer (Agilent 1290/6495 LC-QQQ) equipped with an electrospray ionization source in full scan acquisition mode, and a liquid chromatographic column, the Agilent Infinity Lab Poroshell 120 HILIC-Z (2.1 × 100 mm × 2.7 μm, Agilent), were used to separate water-soluble fractions containing osmoprotectants according to the above conditions. The retention time and peak mass spectra of the samples were compared with the standard. The standard groups used for calibration were divided into maltose, trehalose, proline, glutamic acid, betaine and tetrahydropyrimidine, all procured from Sigma (St. Louis, MO, USA). The amount of total protein in the supernatant was also determined using the Komas Brilliant Blue assay [63]. The number of micrograms of metabolites per milligram of protein in the supernatant was calculated and the solute concentration was expressed as µg mg protein^−1^.

### 4.6. DogH Expression and Purification

Using the reported method [17], the *dogH* gene was cloned from the *D. radiodurans* R1 genome and inserted into the pET28a plasmid between the double-cleaved *BamH* I/*Hind* III, and the recombinant expression vector was transformed into *E. coli* BL21 (DE3) using the primers listed in Table S2. Expression was also induced with 0.5 mM IPTG (isopropyl-2-thiogalactoside) overnight at 16 °C. Bacteria were collected at a low temperature and processed by sonication on ice, and supernatants and precipitates were taken for SDS-PAGE electrophoresis. Protein purification was performed according to the method reported by L. Fredriksen [64]. The DogH protein in the supernatant was further purified and analyzed using a slightly modified method. The samples were subjected to immobilized affinity chromatography using an AKTA pure chromatography system and a Ni2^+^ affinity HisTrap HP 5 mL column (GE HealthCare, Chicago, IL, USA). Protein fractions were analyzed by SDS-PAGE (Bio Rad, Hercules, CA, USA) and fractions containing overexpressed proteins were combined and left to concentrate in ultrafiltration tubes. After concentration, the tubes were replaced with desalting buffer and 5 mM reduced glutathione and 2 mM DTT were added to prevent abnormal signal loss due to oxidation. The purified His-tagged proteins were stored at 4 °C for subsequent studies. SDS-PAGE electropherograms of DogH protein purification are shown in Supplementary Figure S5.

### 4.7. DogH Glycoside Hydrolase Substrate Profiling

In order to analyze the hydrolysis substrate specificity of the DogH glycoside hydrolase, nine carbohydrates with different glycosidic bonds were selected for analysis in this study, as listed in Table 2. According to [17], the method of Benedict’s experiment was used to detect the reducing sugar content of the hydrolyzed substrates. Benedict’s experiment was conducted for a range of concentrations of glucose and maltose to confirm the differences between mono- and disaccharides and to determine reasonable concentrations for the main analysis; the standard curves are shown in Supplementary Figure S6. The final concentrations of reducing and non-reducing sugars were 0.25% and 0.5%, respectively. Amylase decomposition of starch was used as a positive control. Moreover, 200 μg/mL of DogH and an equivalent buffer solution without DogH were added to the solution and incubated at 37 °C for 4 h. The reaction was subsequently terminated by the addition of 500 μL of Benedict’s experiment and incubated at 95 °C for 10 min. The absorbance value of each sample was measured at 320 nm to determine the extent of copper reduction in the substrate. Three replicates of each carbohydrate were measured in the presence and absence of DogH addition.

### 4.8. Statistical Analysis

Unless stated otherwise, experiments were performed three times with similar results. Each bar on the graphs represents the mean of biological replicates, and error bars indicate the SEM (standard error of the mean), as mentioned in the figure legends for each experiment. For all assays, n represents the number of biological replicates. Statistical analysis was performed using GraphPad Prism 8.0. A two-tailed unpaired Student’s t-test with a 95% confidence interval was used to evaluate the difference between two groups. For more than two groups, one-way ANOVA was used. A probability value of *p* ≤ 0.05 was considered significant. Data are presented as averages ± SEM. ****: *p* ≤ 0.0001; ***: *p* ≤ 0.001; **: *p* ≤ 0.01; *: *p* ≤ 0.05; and ns: non-significant.

## 5. Conclusions

In this study, a genus-specific gene, *dogH*, which encodes a novel glycoside hydrolase, was identified in the *D. radiodurans* R1 genome and was associated with the trehalose synthesis pathway by a combined multi-omics approach. Firstly, the biological function of the *dogH* gene was identified and the expression pattern of the gene was examined under different abiotic stress conditions. The expression was upregulated by approximately three times after 1.0 M sorbitol shock for 3 h. The deletion of the *dogH* gene resulted in a strain with reduced resistance to sorbitol stress, indicating that *dogH* can specifically respond to osmotic stress signals and play an important role in the osmotic stress response. Subsequently, GC-MS showed that the maltose and trehalose contents in the wild type were approximately 3.7 times and 7.7 times higher than those in Δ*dogH*, suggesting that the DogH glycoside hydrolase may produce small molecules of honey disaccharide, with the hydrolysis product being maltose. Finally, in vitro experiments showed that the DogH hydrolase specifically hydrolyzes three substances containing α/β-1,4 and α-1,6 glycosidic bonds, namely starch, cellobiose and Melibiose, with the hydrolysis product being maltose. The accumulation of maltose concentration, a precursor of the TreS pathway, mediates the increase in intracellular trehalose content and significantly enhances the resistance of the strain to osmotic stress tolerance.

## Figures and Tables

**Table 1:** Analysis of transcriptional expression of genes related to maltose and trehalose synthesis pathways.

Gene ID	Description	Fold Change
*dogH*(*dr2412*)	Hypothetical protein	2.8
*glgX*(*dr0264*)	1,4-alpha-glucan branching enzyme	9.3
*glgB*(*dr1848*)	1,4-alpha-glucan branching enzyme	10.2
*treY*(*dr0463*)	Maltooligosyl trehalose synthase	5.2
*treZ*(*dr0464*)	Trehalose trehalohydrolase	3.5
*treS*(*dr0933*)	Trehalose synthase	5.7
*dr1141*	Pullulanase/amylase	3.9

**Table 2:** Substrate specificity of DogH.

Carbohydrate	Monosaccharides and Bonds Present
Cellobiose	D-glucose-β-1,4-D-glucose
a-cyclodextrin	Cyclo-[-D-glucose-α-1,4-]6
Lactose	D-galactose-β-1,4-D-glucose
Maltose	D-glucose-α-1,4-D-glucose
Melibiose	D-galactose-α-1,6-D-glucose
Pullulan	[-D-glucose-α-1,4-D-glucose-a-1,4-D-glucose-α-1,6-]n
Starch	[-D-glucose-α-1,4-]n…D-glucose-α-1,6-D-glucose…
Sucrose	D-glucose-α-β-1,2-D-fructose
Trehalose	D-glucose-α-α-1,1-D-glucose

**Table 3:** The list of plasmids and strains.

Plasmid/Strain	Description	Source
pRADZ3	Shuttle vector for *E. coli* and *D. radiodurans*, Chlr (*D. radiodurans*), Ampr (*E. coli*)	Laboratory stock
pKatAPH3	To amplify the kanamycin resistance gene	Laboratory stock
*D. radiodurans* R1	Wild type, served as the strain for generation of the mutants	Laboratory stock
∆*dogH* mutant	*D. radiodurans* with genomic deletion of *dogH* gene *dogH* mutant with pRADZ3 shuttle plasmid introduced into its genome	This study
∆*dogH*-pRADZ3	Complementation of the *dogH* deletion in *D. radiodurans,* transformation of *dogH* mutant with pRADZ3 plasmid	This study
∆*dogH*-com	expressing *D. radiodurans dogH* gene	This study
*WT-dogH*-over	Overexpressing *D. radiodurans dogH* gene	This study
*E. coli* DH5a	The strain expressing the shuttle plasmid pRADZ3	CW Biotech
pET28a(+)	Kan^R^ oripBR322 lacIq T7p	Novagen
*E. coli* BL21	F- ompT hsdSB(rB- mB-) gal dcm(DE3)	TransGen Biotech

## Data Availability

All data underlying the results are included as part of the published article and its Supplementary Materials.

## Supplementary Materials

The following supporting information can be downloaded at: https://www.mdpi.com/article/10.3390/ijms24043437/s1.

## References

[1] IUBMB (1994). Enzyme Nomenclature. Recommendations 1992. Supplement: Corrections and additions. Eur. J. Biochem..

[2] Lombard V., Golaconda Ramulu H., Drula E., Coutinho P.M., Henrissat B. (2014). The carbohydrate-active enzymes database (CAZy) in 2013. Nucl. Acids Res..

[3] van Wyk N., Drancourt M., Henrissat B., Kremer L. (2017). Current perspectives on the families of glycoside hydrolases of *Mycobacterium tuberculosis*: Their importance and prospects for assigning function to unknowns. Glycobiology.

[4] Wolf A., Krämer R., Morbach S. (2003). Three pathways for trehalose metabolism in Corynebacterium glutamicum ATCC13032 and their significance in response to osmotic stress. Mol. Microbiol..

[5] Reina-Bueno M., Argandoña M., Salvador M., Rodríguez-Moya J., Iglesias-Guerra F., Csonka L.N., Nieto J.J., Vargas C. (2012). Role of Trehalose in Salinity and Temperature Tolerance in the Model Halophilic Bacterium Chromohalobacter salexigens. PLoS ONE.

[6] Stam M.R., Danchin E.G.J., Rancurel C., Coutinho P.M., Henrissat B. (2006). Dividing the large glycoside hydrolase family 13 into subfamilies: Towards improved functional annotations of -amylase-related proteins. Protein Eng. Des. Sel..

[7] MacElroy R.D. (1974). Some comments on the evolution of extremophiles. Biosystems.

[8] Banasik M., Stanisławska-Sachadyn A., Hildebrandt E., Sachadyn P. (2017). In vitro affinity of Deinococcus radiodurans MutS towards mismatched DNA exceeds that of its orthologues from *Escherichia coli* and *Thermus thermophilus*. J. Biotechnol..

[9] Blasius M., Sommer S., Hübscher U. (2008). Deinococcus radiodurans: What belongs to the survival kit?. Crit. Rev. Biochem. Mol. Biol..

[10] Slade D., Radman M. (2011). Oxidative stress resistance in Deinococcus radiodurans. Microbiol. Mol. Biol. Rev..

[11] White O., Eisen J.A., Heidelberg J.F., Hickey E.K., Peterson J.D., Dodson R.J., Haft D.H., Gwinn M.L., Nelson W.C., Richardson D.L. (1999). Genome sequence of the radioresistant bacterium Deinococcus radiodurans R1. Science.

[12] Ruhal R., Kataria R., Choudhury B. (2013). Trends in bacterial trehalose metabolism and significant nodes of metabolic pathway in the direction of trehalose accumulation. Microb. Biotechnol..

[13] Chen L., Wei X., Liu G.-L., Hu Z., Chi Z.-M., Chi Z. (2020). Glycerol, trehalose and vacuoles had relations to pullulan synthesis and osmotic tolerance by the whole genome duplicated strain Aureobasidium melanogenum TN3-1 isolated from natural honey. Int. J. Biol. Macromol..

[14] Chi Z., Liu J., Ji J., Meng Z. (2003). Enhanced conversion of soluble starch to trehalose by a mutant of Saccharomycopsis fibuligera sdu. J. Biotechnol..

[15] Zhang L., Lu G., Huang X., Guo H., Su X., Han L., Zhang Y., Qi Z., Xiao Y., Cheng H. (2020). Overexpression of the caryophyllene synthase gene *GhTPS1* in cotton negatively affects multiple pests while attracting parasitoids. Pest Manag. Sci..

[16] Gao L., Zhou Z., Chen X., Zhang W., Lin M., Chen M. (2020). Comparative Proteomics Analysis Reveals New Features of the Oxidative Stress Response in the Polyextremophilic Bacterium Deinococcus radiodurans. Microorganisms.

[17] Bradshaw W.J., Kirby J.M., Roberts A.K., Shone C.C., Acharya K.R. (2017). The molecular structure of the glycoside hydrolase domain of Cwp19 from *Clostridium difficile*. FEBS J..

[18] Weisburg W.G., Giovannoni S.J., Woese C.R. (1989). The *Deinococcus-Thermus* phylum and the effect of rRNA composition on phylogenetic tree construction. Syst. Appl. Microbiol..

[19] Omelchenko M.V., Wolf Y.I., Gaidamakova E.K., Matrosova V.Y., Vasilenko A., Zhai M., Daly M.J., Koonin E.V., Makarova K.S. (2005). Comparative genomics of *Thermus thermophilus* and *Deinococcus radiodurans*: Divergent routes of adaptation to thermophily and radiation resistance. BMC Evol. Biol..

[20] Makarova K.S., Aravind L., Wolf Y.I., Tatusov R.L., Minton K.W., Koonin E.V., Daly M.J. (2001). Genome of the extremely radiation-resistant bacterium Deinococcus radiodurans viewed from the perspective of comparative genomics. Microbiol. Mol. Biol. Rev..

[21] Jordan I.K., Makarova K.S., Wolf Y.I., Koonin E.V. (2001). Gene conversions in genes encoding outer-membrane proteins in *H. pylori* and *C. pneumoniae*. Trends Genet..

[22] Gury J., Barthelmebs L., Tran N.P., Diviès C., Cavin J.-F. (2004). Cloning, deletion, and characterization of PadR, the transcriptional repressor of the phenolic acid decarboxylase-encoding padA gene of Lactobacillus plantarum. Appl. Environ. Microbiol..

[23] Makarova K.S., Omelchenko M.V., Gaidamakova E.K., Matrosova V.Y., Vasilenko A., Zhai M., Lapidus A., Copeland A., Kim E., Land M. (2007). Deinococcus geothermalis: The pool of extreme radiation resistance genes shrinks. PLoS ONE.

[24] Yuan M., Chen M., Zhang W., Lu W., Wang J., Yang M., Zhao P., Tang R., Li X., Hao Y. (2012). Genome sequence and transcriptome analysis of the radioresistant bacterium Deinococcus gobiensis: Insights into the extreme environmental adaptations. PLoS ONE.

[25] Murawska M., Ladurner A.G. (2020). Bromodomain AAA+ ATPases get into shape. Nucleus.

[26] Ogura T., Wilkinson A.J. (2001). AAA+ superfamily ATPases: Common structure--diverse function. Genes Cells.

[27] Pollet A., Delcour J.A., Courtin C.M. (2010). Structural determinants of the substrate specificities of xylanases from different glycoside hydrolase families. Crit. Rev. Biotechnol..

[28] Kirsch F., Klähn S., Hagemann M. (2019). Salt-Regulated Accumulation of the Compatible Solutes Sucrose and Glucosylglycerol in Cyanobacteria and Its Biotechnological Potential. Front. Microbiol..

[29] Empadinhas N., da Costa M.S. (2006). Diversity and biosynthesis of compatible solutes in hyper/thermophiles. Int. Microbiol..

[30] Ash C. (2017). Trehalose confers superpowers. Science.

[31] Argüelles J.C. (2000). Physiological roles of trehalose in bacteria and yeasts: A comparative analysis. Arch. Microbiol..

[32] Paul M.J., Primavesi L.F., Jhurreea D., Zhang Y. (2008). Trehalose Metabolism and Signaling. Annu. Rev. Plant Biol..

[33] Stracke C., Meyer B.H., Hagemann A., Jo E., Lee A., Albers S.-V., Cha J., Bräsen C., Siebers B. (2020). Salt Stress Response of Sulfolobus acidocaldarius Involves Complex Trehalose Metabolism Utilizing a Novel Trehalose-6-Phosphate Synthase (TPS)/Trehalose-6-Phosphate Phosphatase (TPP) Pathway. Appl. Environ. Microbiol..

[34] Zhu Y., Zhang J., Wei D., Wang Y., Chen X., Xing L., Li M. (2008). Isolation and Identification of a Thermophilic Strain Producing Trehalose Synthase from Geothermal Water in China. Biosci. Biotechnol. Biochem..

[35] De Smet K.A.L., Weston A., Brown I.N., Young D.B., Robertson B.D. (2000). Three pathways for trehalose biosynthesis in mycobacteria. Microbiology.

[36] Maruta K., Mitsuzumi H., Nakada T., Kubota M., Chaen H., Fukuda S., Sugimoto T., Kurimoto M. (1996). Cloning and sequencing of a cluster of genes encoding novel enzymes of trehalose biosynthesis from thermophilic archaebacterium Sulfolobus acidocaldarius. Biochim. Biophys. Acta.

[37] Wannet W.J., Op den Camp H.J., Wisselink H.W., van der Drift C., Van Griensven L.J., Vogels G.D. (1998). Purification and characterization of trehalose phosphorylase from the commercial mushroom Agaricus bisporus. Biochim. Biophys. Acta.

[38] Qu Q., Lee S.-J., Boos W. (2004). TreT, a novel trehalose glycosyltransferring synthase of the hyperthermophilic archaeon Thermococcus litoralis. J. Biol. Chem..

[39] Ryu S.-I., Park C.-S., Cha J., Woo E.-J., Lee S.-B. (2005). A novel trehalose-synthesizing glycosyltransferase from Pyrococcus horikoshii: Molecular cloning and characterization. Biochem. Biophys. Res. Commun..

[40] Silva Z., Alarico S., Nobre A., Horlacher R., Marugg J., Boos W., Mingote A.I., da Costa M.S. (2003). Osmotic Adaptation of *Thermus thermophilus* RQ-1: Lesson from a Mutant Deficient in Synthesis of Trehalose. J. Bacteriol..

[41] Krisko A., Radman M. (2013). Biology of Extreme Radiation Resistance: The Way of Deinococcus radiodurans. Cold Spring Harb. Perspect. Biol..

[42] Guan N., Li J., Shin H.-D., Du G., Chen J., Liu L. (2017). Microbial response to environmental stresses: From fundamental mechanisms to practical applications. Appl. Microbiol. Biotechnol..

[43] Mocali S., Chiellini C., Fabiani A., Decuzzi S., de Pascale D., Parrilli E., Tutino M.L., Perrin E., Bosi E., Fondi M. (2017). Ecology of cold environments: New insights of bacterial metabolic adaptation through an integrated genomic-phenomic approach. Sci. Rep..

[44] Im S., Joe M., Kim D., Park D.-H., Lim S. (2013). Transcriptome analysis of salt-stressed Deinococcus radiodurans and characterization of salt-sensitive mutants. Res. Microbiol..

[45] Dani P., Ujaoney A.K., Apte S.K., Basu B. (2017). Regulation of potassium dependent ATPase (kdp) operon of Deinococcus radiodurans. PLoS ONE.

[46] Yu J., Li T., Dai S., Weng Y., Li J., Li Q., Xu H., Hua Y., Tian B. (2017). A tamB homolog is involved in maintenance of cell envelope integrity and stress resistance of Deinococcus radiodurans. Sci. Rep..

[47] Liu Y., Zhang C., Wang Z., Lin M., Wang J., Wu M. (2021). Pleiotropic roles of late embryogenesis abundant proteins of Deinococcus radiodurans against oxidation and desiccation. Comput. Struct. Biotechnol. J..

[48] Alsheikh M.K., Heyen B.J., Randall S.K. (2003). Ion binding properties of the dehydrin ERD14 are dependent upon phosphorylation. J. Biol. Chem..

[49] Jiang S., Wang J., Liu X., Liu Y., Guo C., Zhang L., Han J., Wu X., Xue D., Gomaa A.E. (2017). DrwH, a novel WHy domain-containing hydrophobic LEA5C protein from Deinococcus radiodurans, protects enzymatic activity under oxidative stress. Sci. Rep..

[50] Awile O., Krisko A., Sbalzarini I.F., Zagrovic B. (2010). Intrinsically disordered regions may lower the hydration free energy in proteins: A case study of nudix hydrolase in the bacterium Deinococcus radiodurans. PLoS Comput. Biol..

[51] Oren A. (2008). Microbial life at high salt concentrations: Phylogenetic and metabolic diversity. Saline Syst..

[52] Alarico S., Empadinhas N., Simões C., Silva Z., Henne A., Mingote A., Santos H., da Costa M.S. (2005). Distribution of genes for synthesis of trehalose and Mannosylglycerate in *Thermus* spp. and direct correlation of these genes with halotolerance. Appl. Environ. Microbiol..

[53] Santos H., da Costa M.S. (2002). Compatible solutes of organisms that live in hot saline environments. Environ. Microbiol..

[54] Agapov A.A., Kulbachinskiy A.V. (2015). Mechanisms of stress resistance and gene regulation in the radioresistant bacterium Deinococcus radiodurans. Biochem. Mosc..

[55] Wang L., Xu G., Chen H., Zhao Y., Xu N., Tian B., Hua Y. (2008). DrRRA: A novel response regulator essential for the extreme radioresistance of Deinococcus radiodurans. Mol. Microbiol..

[56] Xu G., Wang L., Chen H., Lu H., Ying N., Tian B., Hua Y. (2008). RecO is essential for DNA damage repair in Deinococcus radiodurans. J. Bacteriol..

[57] Almagro Armenteros J.J., Tsirigos K.D., Sønderby C.K., Petersen T.N., Winther O., Brunak S., von Heijne G., Nielsen H. (2019). SignalP 5.0 improves signal peptide predictions using deep neural networks. Nat. Biotechnol..

[58] Kumar S., Stecher G., Tamura K. (2016). MEGA7: Molecular Evolutionary Genetics Analysis Version 7.0 for Bigger Datasets. Mol. Biol. Evol..

[59] Hong Y., Guo M., Wang J. (2021). ENJ algorithm can construct triple phylogenetic trees. Mol. Ther. Nucleic Acids.

[60] Biasini M., Bienert S., Waterhouse A., Arnold K., Studer G., Schmidt T., Kiefer F., Gallo Cassarino T., Bertoni M., Bordoli L. (2014). SWISS-MODEL: Modelling protein tertiary and quaternary structure using evolutionary information. Nucleic Acids Res..

[61] Guo L., Zhao M., Tang Y., Han J., Gui Y., Ge J., Jiang S., Dai Q., Zhang W., Lin M. (2021). Modular Assembly of Ordered Hydrophilic Proteins Improve Salinity Tolerance in *Escherichia coli*. Int. J. Mol. Sci..

[62] Chen Y., Zhao M., Lv M., Lin M., Wang J., Zuo K. (2022). A Novel Small RNA, DsrO, in Deinococcus radiodurans Promotes Methionine Sulfoxide Reductase (msrA) Expression for Oxidative Stress Adaptation. Appl. Environ. Microbiol..

[63] Bradford M.M. (1976). A rapid and sensitive method for the quantitation of microgram quantities of protein utilizing the principle of protein-dye binding. Anal. Biochem..

[64] Fredriksen L., Stokke R., Jensen M.S., Westereng B., Jameson J.-K., Steen I.H., Eijsink V.G.H. (2019). Discovery of a Thermostable GH10 Xylanase with Broad Substrate Specificity from the Arctic Mid-Ocean Ridge Vent System. Appl. Environ. Microbiol..

