# Supplementary material for: A Novel Glycoside Hydrolase DogH Utilizing Soluble Starch to Maltose Improve Osmotic Tolerance in Deinococcus radiodurans
Source: Int J Mol Sci. 2023 Feb 8;24(4):3437. doi: 10.3390/ijms24043437 (PMC9967864; doi:10.3390/ijms24043437)
Supplement: Supplementary file 1 [file ijms-24-03437-s001.zip › Supplementary Material-ijms 2153743.pdf]

Supplementary Material

A Novel Glycoside Hydrolase DogH Utilizing Soluble Starch to Maltose Improve Osmotic Tolerance in *Deinococcus radiodurans*

Yuan Gui [1,2,3], Min Lin [1,2] , Yongliang Yan[2] , Shijie Jiang[1], Zhengfu Zhou[2,*] and Jin Wang [1,2,*]

1 College of Life Science and Engineering, Southwest University of Science and Technology, Mianyang 621000, China.
2 Key Laboratory of Agricultural Microbiome (MARA), Biotechnology Research Institute, Chinese Academy of Agricultural Sciences, Beijing 100081, China.
* Correspondence: zhouzhengfu@caas.cn (Z.Z.); wangjin@caas.cn (J.W.)

Table S1 .List of 24 small molecules of sugars

Number	Name	Molecular weight	Molecular formula
1	D-Lyxose	150.1299	$C_5H_{10}O_5$
2	D-(-)-Arabinose	150.13	$C_5H_{10}O_5$
3	D-ribose	150.13	$C_5H_{10}O_5$
4	D-(+)-Fucose	164.16	$C_6H_{12}O_5$
5	L-Rhamnose	182.11	$C_6H_{14}O_6$
6	Adonitol	152.15	$C_5H_{12}O_5$
7	D-xylose	150.13	$C_5H_{10}O_5$
8	L-(-)-Sorbose	180.16	$C_6H_{12}O_6$
9	D(+)-Mannose	360.3118	$C_{12}H_{24}O_{12}$
10	D-Mannitol	182.17	$C_6H_{14}O_6$
11	D(-)-Fructose	180.1559	$C_6H_{12}O_6$
12	D-galactose	180.1559	$C_6H_{12}O_6$
13	D(+)-Glucose	180.16	$C_6H_{12}O_6$
14	Dulcitol	182.1718	$C_6H_{14}O_6$
15	D-Fructose-1,6-diphosphate	340.12	$C_6H_{14}O_{12}P_2$
16	Xylobiose	282.24	$C_{10}H_{18}O_9$
17	D(+)-Sucrose	342.2965	$C_{12}H_{22}O_{11}$
18	Maltose	360.32	$C_{12}H_{22}O_{11}\cdot H_2O$
19	Turanose	342.2965	$C_{12}H_{22}O_{11}$
20	D-Glucopyranose	342.2965	$C_{12}H_{22}O_{11}$
21	D(+)-Cellobiose	342.2965	$C_{12}H_{22}O_{11}$
22	Laminaribiose	342.3	$C_{12}H_{22}O_{11}$
23	Melibiose	342.30	$C_{12}H_{22}O_{11}$
24	Gentiobiose	342.30	$C_{12}H_{22}O_{11}$

Table S2 List of primers used in this study

**Gene/Fragment name**	**Sequence (5′–3′)**
U-F（P1）	TGACATCCGTGCCCACCCGATG
U-R（P2）	GTTTTTCTAATCAGGATCCTCTAGCGGGTCCGCTGCCGGAGGCGCCT
K-F（P3）	AGGCGCCTCCGGCAGCGGACCCGCTAGAGGATCCTGATTAGAAAAAC
K-R（P4）	GTGCTCAGGTAGCTCAGGCGCCACGGTATCGATAAGCTTGATAT
D-F（P5）	ATATCAAGCTTATCGATACCGTGGCGCCTGAGCTACCTGAGCAC
D-R（P6）	GGACGTTCGCGGACCGCGTGTA
YZ-F（P7）	TTGCCTGGGCGAGCGTCACC
YZ-R（P8）	AATGGGTACGCGACGGCCTG
YZ-UD-F（P9）	AGCCGGTGACGGTGGGCGCCGCTCA
YZ-UD-R（P10）	TGCGCGGTGGGTTTTCTGCTCGCT
28a-dogH-F（P11）	GTGGACAGCAAATGGGTCGCGGATCCATGCCTGCTCCACCAGCACTCG
dogH-28a-R（P12）	TGGTGCTCGAGTGCGGCCGCAAGCTTTCAGCGCTTCCCCGGCTTCGTG
Z3-dogH-F（P13）	ACACTGGCGGCCGTTACTAGTTGCTGGACGACGTGGAAGCGAGC
dogH-Z3-R（P14）	ATGCCTGCAGGTCGAATCGGATCCTCAGCGCTTCCCCGGCTTCGTGGGT
Q16S-F	ATTCCTGGTGTAGCGGTG
Q16S-R	CATCGTTTAGGGTGGAC
QdogH-F	AGACGGTGAACGACGCAGTG
QdogH-R	TTGGTGACGATGGCGAGGG
Q0264-F	GCCGTATGCCTCCATCAACT
Q0264-R	GGTGATGTTGTGGTTGTGGC
Q1141-F	GCAGACCTGGGACCACGACA
Q1141-R	GCAGTTGAAGGCCACATACGC
Q1848-F	GCTATCAGGTCACGGGCTAC
Q1848-R	GCTATCAGGTCACGGGCTAC
QTreY-F	CTGCTACACGAAACCGACCT
QTreY-R	CGCTTTTTGCAAGTAGGCGT
QTreZ-F	ACTGCGTGTTCTACGAGGTG
QTreZ-R	GCATCACTTGAATGGCCGTC
QTreS-F	TACTACGGCGACGAAATCGG
QTreS-R	TCCTGAATCGGCGGGAAAAA

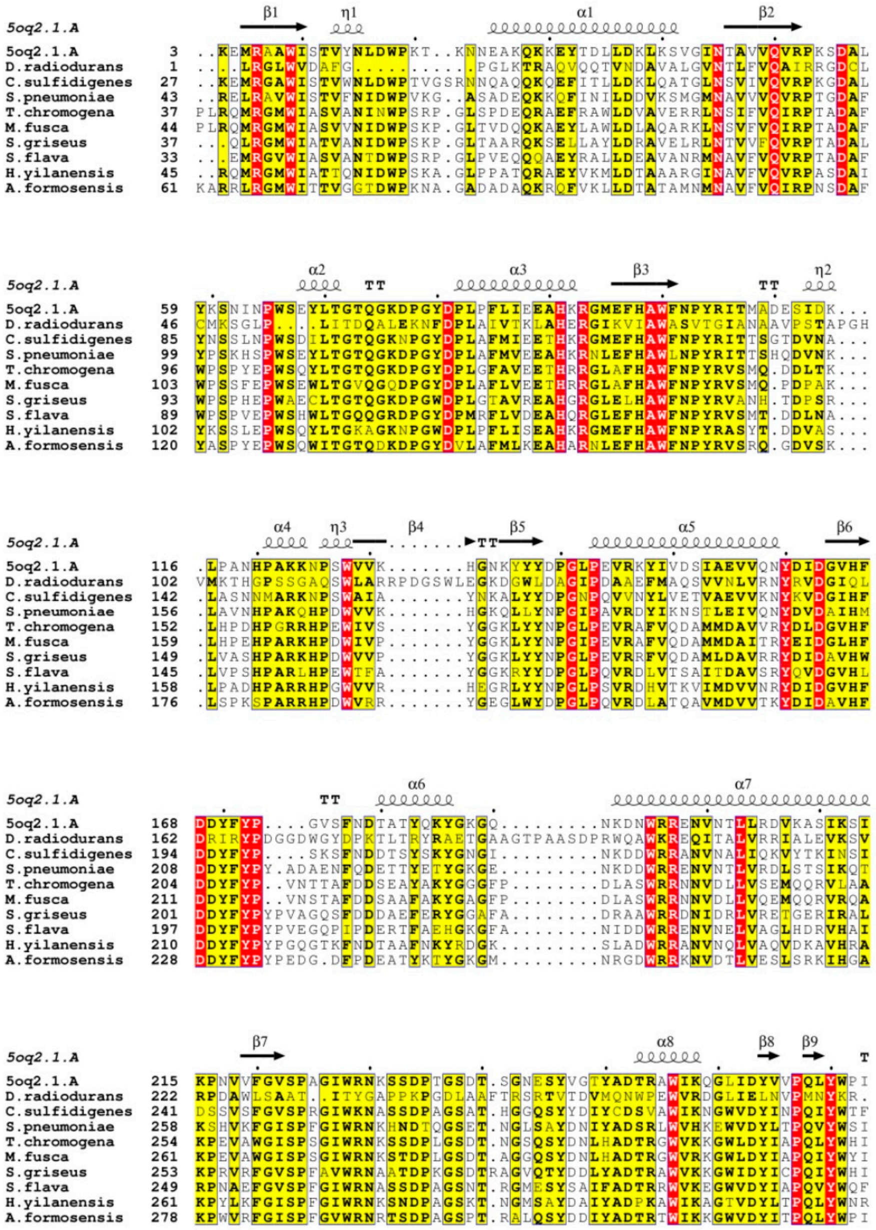

Figure S1. Predicted structure and multiple sequence alignment of *DogH*$_{62\text{-}339}$ to GHL10 proteins. Note: *DogH* residue numbering is given under the alignment and secondary structure is given below. The eight β-strands and *α*-helices that form the TIM barrel are indicated, other *α*-helices and β-strands are not labelled, 3_{10} helices and b-bridges are also indicated with zig-zag patterns and small arrows respectively. Residues conserved across all sequences are highlighted in red, moderately conserved residues in yellow, no conserved residues in grey. Species and NCBI references are as follows: *Clostridium sulfidigenes*, WP_035134795.1; *Streptococcus pneumoniae*, COD97312.1; *Thermomonospora chromogena*, SDQ39562.1; *Microtetraspora fusca*, WP_066947673.1; *Streptomyces griseus*, WP_030738522.1; *Saccharopolyspora flava*, SFT07258.1; *Herbidospora yilanensis*, WP_062357460.1; *Actinomadura formosensis*, WP_067802682.1.

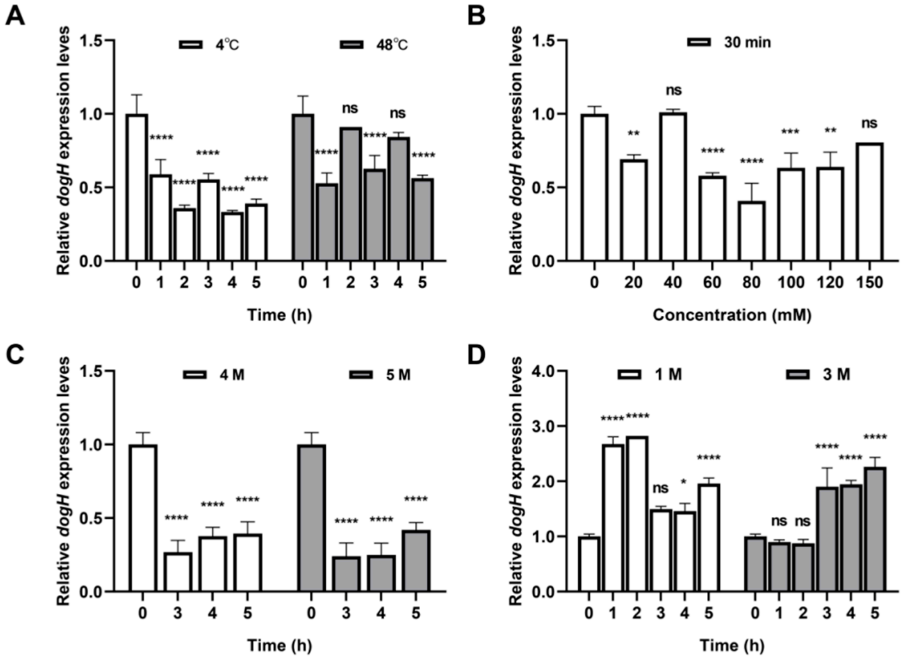

Figure S2. Induced expression analysis of *dogH* gene under abiotic stress. A-D: Relative expression levels of *dogH* in response to the treatments of heat (48℃) (A), cold (4℃) (A) ,H_2O_2 (B), NaCL (C) ,sorbitol(D) in *D. radiodurans*. Asterisks indicate statistically significant difference of the value compared to that of untreated cells. One−way ANOVA and Dunnett's multiple−comparison test;. A probability value of $p \leq 0.05$ was considered significant. Data are presented as averages ± SEM. ****: $p \leq 0.0001$; ***: $p \leq 0.001$; **: $p \leq 0.01$; *: $p \leq 0.05$; and ns: non−significant.

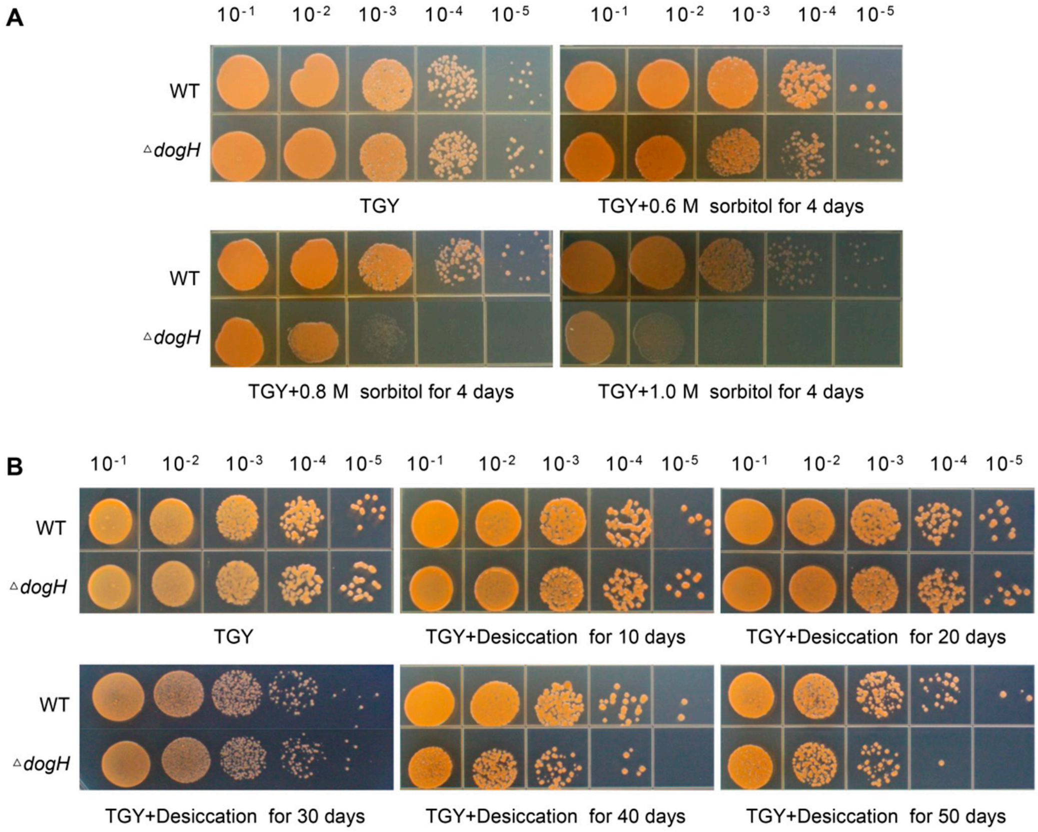

Figure S3. The knockout of *dogH* gene has an effect on the tolerance to abiotic stress. (A): Phenotype of different *D. radiodurans* strains under the treatments of sorbitol stress. Left images: untreated control; Right images: different abiotic stress treatments. (B): Phenotype of different *D. radiodurans* strains under the treatments of desiccation stress. WT: wild type strain, Δ *dogH*: *dogH* deleted mutant.

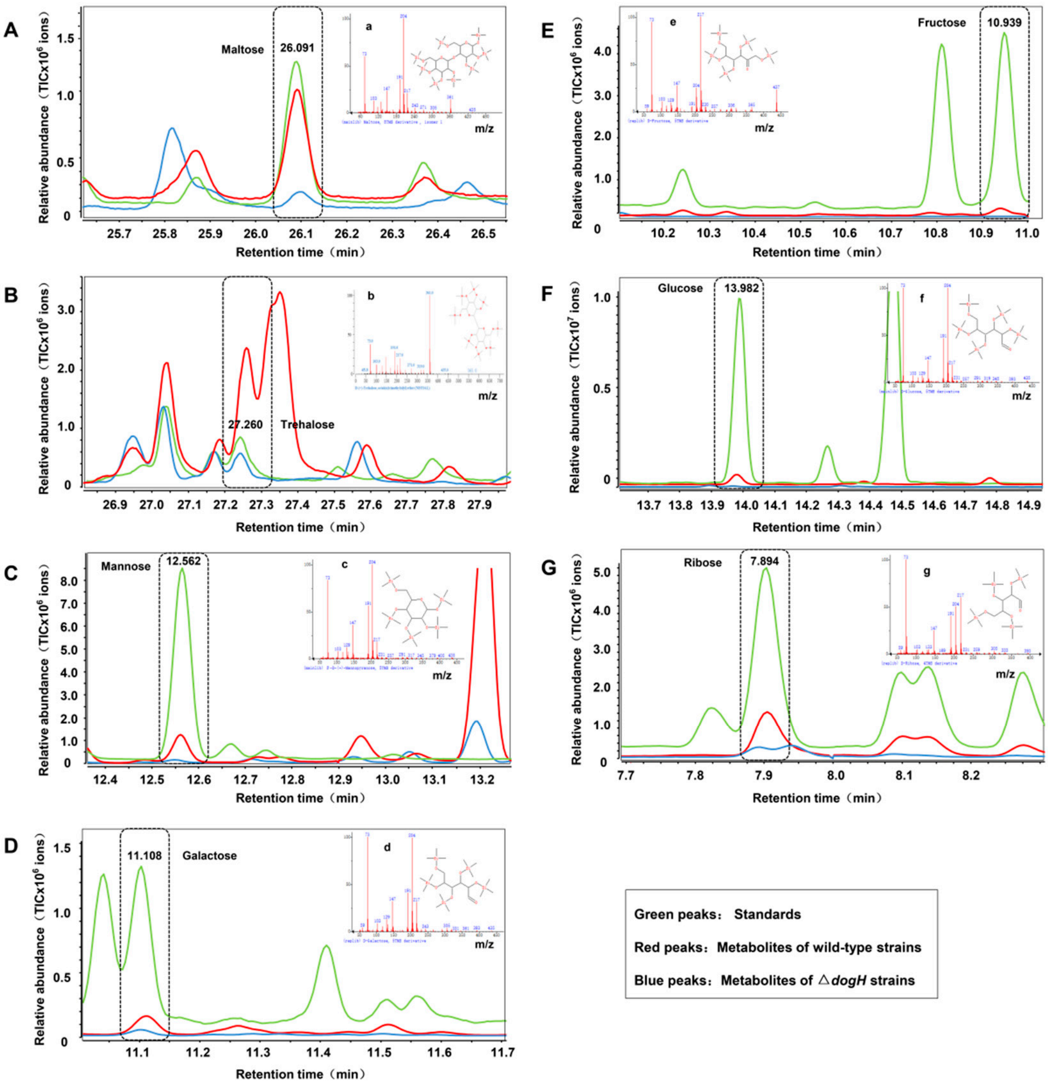

Figure S4. GC-MS analysis of *D. radiodurans* different strains metabolites under sorbitol stress. DR wild-type and *ΔdogH*, Figure A-G: Maltose；Trehalose；Mannose；Galactose；Fructose；Glucose；Ribose, respectively.

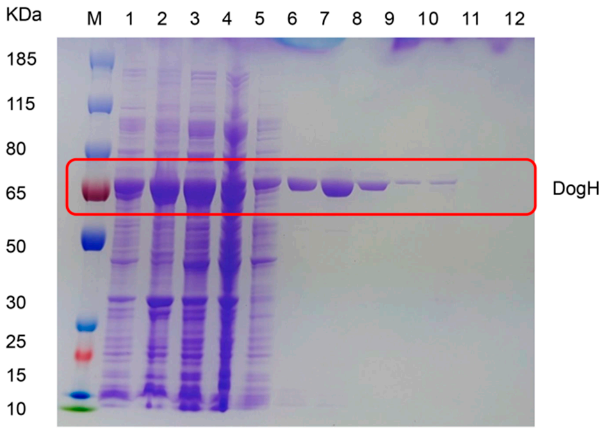

Figure S5. SDS-PAGE analysis of purified DogH. Note: Next to a lane with size marker proteins the gel shows lanes with purified full-length enzyme .Cultures were incubated with 0.5 mM IPTG at 16°C overnight. Lane M, molecular weight standards (DogH; predicted size 61.25kDa).

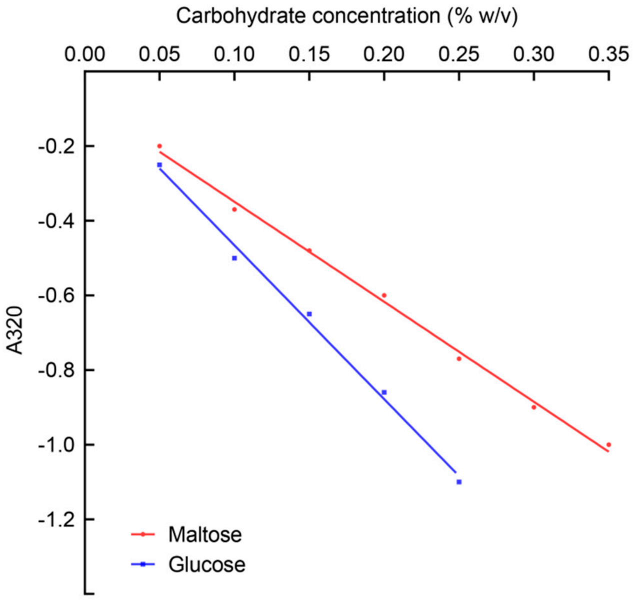

Figure S6. Standard curve of Benedict’s assay. Note: The Benedict's test can be used to differentiate between the conformations of monosaccharides and disaccharides. Glucose significantly reduced the A320 content more than an equivalent concentration of maltose.

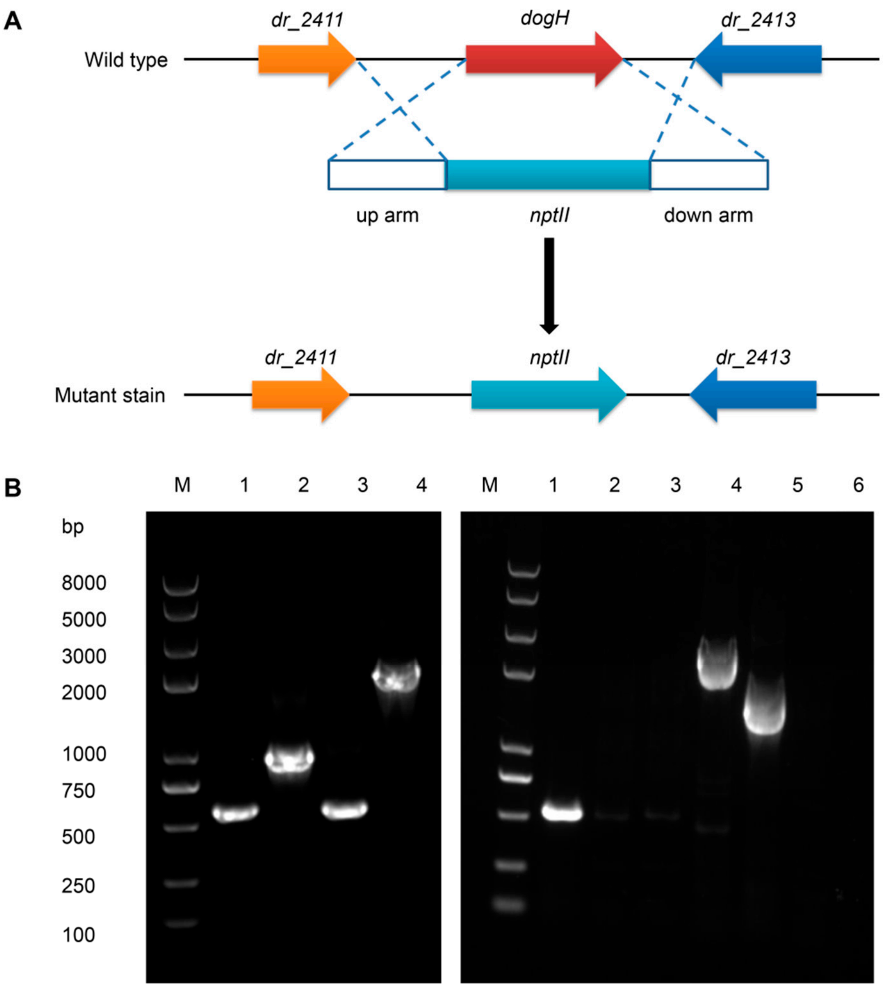

Figure S7. Construction and verification of the Δ*dogH* mutant. (A) Schematic representation of the Δ*dogH* mutant generated by replacing the *dogH* region with the kanamycin resistance gene *nptII* (Kan^R). (B) PCR verification of the Δ*dogH* mutant. Lane M: Trans2K PlusII DNA marker; left lanes 1-4: PCR products amplified from the U, K, D and UKD. Right lanes 1&4: PCR products amplified from the Δ*dogH* mutant using primers P7/P8 and P9/P10, respectively; lanes 2 & 5: PCR products amplified from DR−WT using primers P7/P8 and P9/P10, respectively; and lanes 3 & 6: PCR products amplified from sterile water using primers P7/P8 and P9/P10, respectively.
